# Phylogeny, structural evolution and functional diversification of the plant *PHOSPHATE1* gene family: a focus on *Glycine max*

**DOI:** 10.1186/1471-2148-13-103

**Published:** 2013-05-24

**Authors:** Lingli He, Man Zhao, Yan Wang, Junyi Gai, Chaoying He

**Affiliations:** 1 State Key Laboratory of Systematic and Evolutionary Botany, Institute of Botany, Chinese Academy of Sciences, Nanxincun 20, Xiangshan, 100093 Beijing, China; 2 University of Chinese Academy of Sciences, Yuquan Road 19, 100049, Beijing, China; 3 Soybean Research Institute/National Center for Soybean Improvement/MOA Key Laboratory of Biology and Genetic Improvement of Soybean/National Key Laboratory for Crop Genetics and Germplasm Enhancement, Nanjing Agricultural University, 210095, Nanjing, China

**Keywords:** Evolution, Gene expression, *PHO1* gene family, Phylogeny, Soybean

## Abstract

**Background:**

*PHOSPHATE1* (*PHO1*) gene family members have diverse roles in plant growth and development, and they have been studied in *Arabidopsis*, rice, and *Physcomitrella*. However, it has yet to be described in other plants. Therefore, we surveyed the evolutionary patterns of genomes within the plant *PHO1* gene family, focusing on soybean (*Glycine max*) due to its economic importance.

**Results:**

Our data show that *PHO1* genes could be classified into two major groups (Class I and Class II). Class I genes were only present and expanded in dicotyledonous plants and *Selaginella moellendorffii*; Class II genes were found in all land plants. Class I sequence losses in other lineages may be attributed to gene loss after duplication events in land plant evolution. Introns varied from 7 to 14, and ancestral state reconstruction analyses revealed that genes with 13 introns were ancestral, thus suggesting that the intron loss was a chief constituent of *PHO1* gene evolution. In the soybean genome, only 12 *PHO1*-like genes (*GmaPHO1*) were detected at the mRNA level. These genes display tissue-specific or tissue-preferential expression patterns during soybean plant and fruit development. Class I genes were more broadly expressed than Class II. *GmaPHO1* genes had altered expression in response to salt, osmotic, and inorganic phosphate stresses.

**Conclusions:**

Our study revealed that *PHO1* genes originated from a eukaryotic ancestor and that two major classes formed in land plants. Class I genes are only present in dicots and lycophytes. *GmaPHO1*genes had diverse expression patterns in soybean, indicating their dramatic functional diversification.

## Background

*PHOSPHATE1* (*PHO1*) genes play diverse roles in plant growth and development; for example, some are important for inorganic phosphate (Pi) sensing and transport. *Arabidopsis thaliana AthPHO1* (At3g23430), the first species in which the *PHO1* gene family was identified, was reported to be key for long-distance transfer of Pi from the root to the shoot [1,2], as well as signal transduction of the long-distance Pi-deficiency response [3,4]. *AthPHO1* is predominantly expressed in root vascular tissues and is involved in root Pi loading into the apoplastic space of the xylem vessels [1]. Gene ectopic expression of *AthPHO1* in leaves [5] and in mesophyll protoplasts [6] mediates cellular specific phosphate efflux. The *Arabidopsis* genome encodes 10 additional genes (identified as *AthPHO1; H1* to *AthPHO1; H10*) which are homologous to *AthPHO1*[7]. However, only *AthPHO1; H1* complements *athpho1* mutants when expressed under control of the *AthPHO1* promoter, indicating that only these two members are involved in long-distance Pi transport from root to shoots [8]. The rice genome has only three *PHO1* homologs (*OsaPHO1; H1, H2, H3*) clustering with *AthPHO1* and *AthPHO1; H1*, but only *OsaPHO1; H2* complements *athpho1* mutants, suggesting a role in phosphate homeostasis [9].

Seven *PHO1*-like genes are present in the non-vascular land plant *Physcomitrella patens* genome; however these gene functions are unclear [10]. In *Arabidopsis*, some *PHO1* homologues are also expressed in non-vascular tissues such as hydathodes, trichomes, and pollen grains [7]. *AthPHO1; H4* plays a role in the response of hypocotyls to blue light [11], seed size and flowering [12-14]. *AthPHO1; H10* was reported to be induced by numerous stresses, including local wounding responses [15,16]. Recently, *AthPHO1* has been suggested to play an important role in the stomatal response to abscisic acid (ABA); its expression in guard cells is induced by ABA treatment [17]. Therefore, plant *PHO1* genes have undergone a functional diversification and acquired diverse roles beyond Pi transport and homeostasis. Furthermore, the PHO1 homolog xenotropic and polytropic retrovirus receptor 1 (XPR1) in mammals functions as a receptor [18]; and the suppressor of yeast Gα protein deletion (SYG1) protein is involved in the mating pheromone signal transduction pathway [19]. Such functions suggest that *PHO1* genes have diversified over time.

Soybean (*Glycine max*) is a crop of economic significance and a world-wide source of high quality protein and vegetable oils [20]. Understanding mechanisms of nutrient homeostasis regulation, especially Pi, may increase soybean yield. *PHO1* gene family molecular evolution and functional diversification remains understudied, so whether soybean *PHO1* genes are involved in Pi sensing and transfer is unclear. To address this gap in the scientific literature, we experimentally characterized *PHO1* genes of *G. max* (*GmaPHO1*) in Nannong1138-2 cultivars, and investigated their evolutionary patterns on a broad-scale phylogenetic framework. Soybean is a diploidized ancient tetraploid species [21,22], and *GmaPHO1* genes might have distinct evolutionary patterns that differ from their orthologs in other plant species. *GmaPHO1* genes may have unique expression patterns in response to various stimuli such as Pi stress, contributing to plant diversification. Our detailed gene expression analysis revealed that these genes may be important for plant organ development and responses to various abiotic stimuli.

## Results

### Phylogenetic analysis of the plant *PHO1* genes

Phylogenetic analyses allow us to identify evolutionary conservation and divergence of genes. To understand the evolution of the plants *PHO1* gene family, we used *AthPHO1* to query the NCBI and Phytozome databases (see Methods). We obtained 223 of *PHO1*-like sequences from 32 plant species, including two chlorophyta species, one moss, one lycophyte, two gymnosperms, and 26 angiosperms. Whole-genome sequences of most plant species are available, except for the two gymnosperms (see Additional file 1: Table S1). Currently, 6 chlorophyta genomes were released; however, only two *PHO1* genes were found in two species of *Ostreococcus lucimarinus* and *Micromonas pusilla RCC299*. We observed that few homologs had evolved in yeast and animals; however, no *PHO1*-like sequences were found in prokaryotes, suggesting that *PHO1*-like genes originated in a common eukaryotic ancestor.

**Figure 1:**
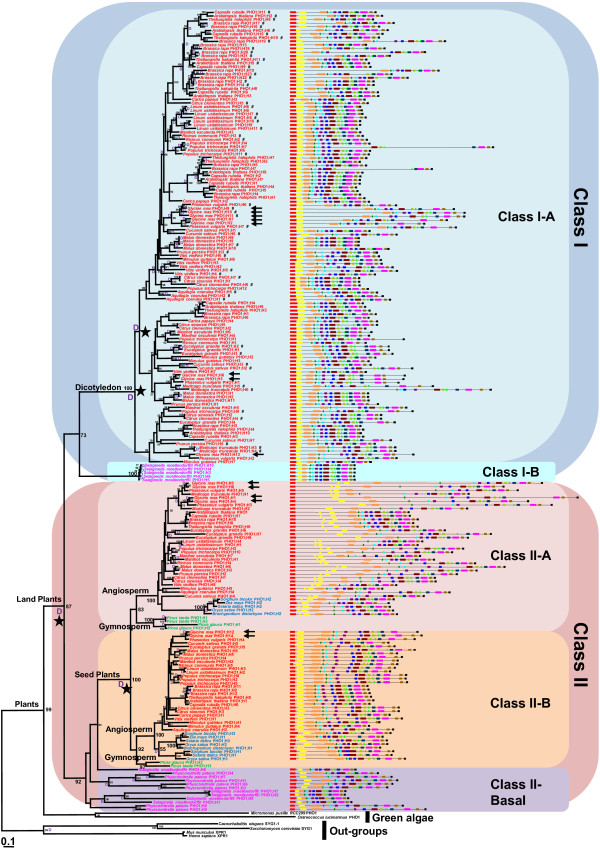
**Phylogenetic tree and gene structure of the *PHO1 *gene family in plants.** The maximum likelihood tree of the land plant *PHO1* genes constructed based on the deduced full-length peptide amino acid sequences using the proteins SYG1 in *Saccharomyces cerevisiae*, SYG-1 in *Caenorhabditis elegans*, and XPR1in *Mus musculus* and *Homo sapiens* as out-groups. Bootstrap values (>50%) for this tree are shown on each branch. The different classes are marked with different colored backgrounds. The genes from the basal land plants are indicated in rose pink; dicot in red, monocots in blue and gymnosperm in green, respectively. The *PHO1*-like genes in soybean are indicated by arrows. The symbol ‘D’ indicates the detected gene duplication events, and the symbol ‘#’ indicates genes resulted from tandem duplication in their hosts. The black star indicates the 4 important major duplication events during the expansion of *PHO1* genes in land plants. Gene structure was predicted based on the sequences from the Phytozome database. Colored boxes represent exons and connecting lines indicate the introns, respectively. The structure of *PHO1* genes in the gymnosperm *Pinus taeda* and *Picea glauca* are not represented for lack of their genome sequences.

A maximum likelihood tree was generated using amino acid sequences of the deduced full-length peptides (733 aligned positions) with the best-fit evolutionary JTT (Jones, Taylor and Thornton) model (Figure 1). The WAG (Whelan and Goldman) and LG (Le and Gascuel) models were also tested (see Methods), and resultant tree topologies were consistent with the JTT model; only a few gene relationships were varied within each Class (see Additional file 2: Figure S1). Proteins SYG1 in *Saccharomyces cerevisiae*, SYG-1 in *Caenorhabditis elegans*, and XPR1 in *Mus musculus* and *Homo sapiens* were used as out-groups in phylogenetic analyses. *PHO1* genes from algae formed the basal lineage, whereas *PHO1*-like genes from land plants were monophyletic, apparently forming two major groups (Classes I, II) based on the current tree topology, with a well-supported bootstrap value (87%; Figure 1). One hundred twenty-five *PHO1*-like genes from dicots were specifically assigned to Class IA (with 100% bootstrap value support) with a basal lineage (Class IB) containing 5 sequences from the basal land plants *Selaginella moellendorffii*. In contrast, 91 members were present in Class II, which could be subdivided into Class IIA (41 genes) and Class IIB (38 genes) with basal grade (Class II-Basal) that was a non-monophyletic group including 5 *Selaginella* genes and 7 *Physcomitrella* genes. Class IIA and Class IIB consisted of the sequences from all seed plants with a 100% bootstrap value.

Gene family copy number varied from 2 to 23 among the species, which was not correlated with the genome size (R=−0.15, *P*=0.57) but slightly correlated with number of whole-genome duplication events (WGD) occurring within Viridiplantae (R=0.51, *P*=0.04, see Additional file 3: Table S2) [23-25]. *Brassica rapa* had the largest *PHO1* gene family (23 genes) with a 500 Mb genome size and 4 WGD events. *Brachypodium distachyon* and *Zea mays* had only 2 genes (355 Mb and 2400 Mb genome sizes) with 2 and 3 WGD events occurring. Class I contained 125 genes from 21 dicots and 5 genes from *S. moellendorffii*, while Class II had only 91 genes from all 30 land plants studied (Figure 1; see Additional file 3: Table S2), indicating that the *PHO1* gene family asymmetrically evolved between Class I and Class II.

To investigate *PHO1* gene family expansion and gene diversification, a gene tree was reconciled with the species tree. Analyses revealed 90 duplications and 29 losses, with 164 of the D/L score (duplication events =‘D’; four major duplication events for gene family expansion in land plants are indicated by stars; Figure 1). Class I and II diverged from ancestral duplications, and subclass Class IA arose from at least two major duplication events. Class IIA (close to the well-known *AthPHO1*) and Class IIB (close to the *AthPHO1; H1*) arose from one duplication event occurring in seed plant ancestors, and each class could be divided further into angiosperm and gymnosperm lineages. Class I genes in the dicots and *S.moellendorffii* were preserved and may have resulted from gene loss after the first duplication in other lineages. Inspection of the physical chromosomal location of *PHO1* genes suggested that tandem duplication may have contributed to evolution of the Class I *PHO1* gene family in the specific host. Fifty-five of the Class IA 125 members appeared on native host chromosomes as 2 to 3 gene tandem repeats (highlighted by ‘#’ in Figure 1; see Additional file 4: Table S3).

### Gene structure variation throughout plant *PHO1* gene family

Comparisons of 215 cDNA sequences and genomic DNA from 28 species revealed exons and introns number and position for each individual gene in land plants (Figure 1; see Additional file 5: Table S4 and Additional file 6: Figure S2). Six *PHO1*-like transcripts from gymnosperms were not included; their gene sequences were unavailable. Two *PHO1* gene sequences from green algae had no introns, and diverse gene structures in this family resulted from multiple intron losses and rare intron gains in land plants. Intron numbers of these genes varied from 7 to 14, with widely divergent lengths. All genes in the Class II-basal grade had more than 9 introns, a characteristic that seemed to be maintained during seed plant evolution. Around 87.0% of Class I members and 93.0% of the genes in Class IIA and IIB had more than 9 introns; genes from Class IIA had more than 11 introns, and 73.0% of the *PHO1* genes within Class IIA had 14 introns.

**Figure 2:**
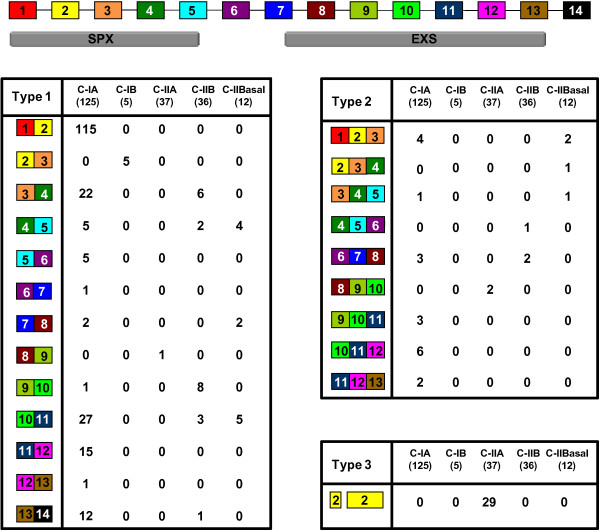
**Structural evolutions of plant *PHO1 *genes.** Variation in exon and intron structure of plant *PHO1* genes. The ancient gene structure with 14-*EXONS* is given. Boxes in different colors indicate different exon and the lines represent introns. Different types of intron loss or intron gain processes in *PHO1* gene family are listed below the 14-exon structure.

To understand *PHO1* gene structures evolution, we reconstructed the ancestral states of exon patterns in 28 land plants excluding gymnosperms (see Additional file 6: Figure S2). Data show that 14-exon structure of *PHO1*genes could represent ancestral land plants structure. Diverse structures of other members in the *PHO1* gene family could be traced back to the 14-exon structure, and their ancestral components could be clearly distinguished. Given that all genes were 14 putative *EXON*s, exons from 4 to13 were highly conserved with respect to lengths and sequences within this family. These exons were observed to encode part of the SPX (Pfam PF03105) domain and the entirety of the EXS (Pfam PF03124) domain (Figure 2), domains which are characteristic of the PHO1 protein family [26-28]. *PHO1* gene structure diversity apparently resulted from intron losses and novel intron gains after the origin of the 14-exon structure (see Additional file 5: Table S4).

Intron loss, as a major molecular event, happened twice at most in the consequent and adjacent exons in *PHO1* land plants genes evolution, whereas novel intron gain was unique to Class IIA. Thus, the divergent patterns of intron number variations could be summarized into three types (Figure 2). Type 1 included the exon-fusion events that happened once in adjacent exons; Type 2 included exon-fusion events that occurred twice in the adjacent exons (involving the three consequent exons), while Type 3 represented a novel intron gain event. Among the 215 genes studied, the exon-fusion events occurred 243 times in pattern Type 1, 28 times in pattern Type 2 and 29 times in pattern Type 3, suggesting that intron loss was main molecular event occurring during evolution of this gene family. In pattern Type 1, we observed 13 exon-fusion events which were location dependent. Such intron loss could occur multiple times (maximally 4 times) on one gene. Pattern Type 2 consisted of 9 events that were location-specific and could occur with Type 1 pattern on one gene. Intron gains (Type 3) occurred 29 times in exon 2 only of Class IIA in 37 genes. We also assessed the intron phase for all *PHO1* genes (see Additional file 6: Figure S2). Intron phases of adjacent exons were virtually conserved in these differently classed genes irrespective of how introns were lost or gained within the 14-exon structure.

### Genomic identification of the *PHO1* gene family in soybean

Release of *Glycine max* (Williams 82) draft soybean genome [22] facilitated the isolation of *GmaPHO1* genes in this investigation. Data-mining using 11 *Arabidopsis PHO1* homologs as queries revealed 14 putative *PHO1* homologous genes (designated as *GmaPHO1; H1* to *GmaPHO1; H14*) in the soybean genome. *GmaPHO1; H2, H3, H6, H7, H9, H10, H11* and *H13* belong to Class Ι; *GmaPHO1; H1, H4, H5* and *H8* are from Class IIA and *GmaPHO1; H12* and *H14* consist of Class IIB (Figure 1, highlighted by arrows). These genes were found to be distributed on 7 of the 20 chromosomes (Table 1; see Additional file 7: Figure S3), and the gene number on each chromosome varied widely, one gene on each chromosome of 1, 7, 9, and 18; two on chromosome 10, three on chromosome 2, and 5 genes were localized to chromosome 20. The number of introns varied from 12 to 14 (Figure 1; see Additional file 8: Table S5). Encoded proteins were predicted to contain the SPX and EXS domains (see Additional file 9: Figure S4), and were estimated to be located on plasma membranes. Each GmaPHO1 protein contained at least 5 trans-membrane segments (Table 1), suggesting the potential function of these proteins as transporters of phosphate absorbed from the soil. GmaPHO1-like proteins range from 753 to 817 amino acids in length and have 30-94% overall identities (Table 1; see Additional file 10: Table S6). Interestingly, several pairs of *GmaPHO1*-like genes on different chromosomes showed high identities in protein sequences, for example, there was 94% identity between GmaPHO1; H1 and GmaPHO1; H4, 93% between GmaPHO1; H12 and GmaPHO1; H14 and 91% between GmaPHO1; H2 and GmaPHO1; H7. These data reflected genome duplication during the soybean evolution [29,30]. The open reading frame (ORF) sequence of these genes was confirmed by isolation of the cDNA from soybean Nanong1138-2. Gene-specific primers were designed according to the predicted sequences from soybean Williams 82 (see Additional file 11: Table S7). Ultimately we obtained 12 of the 14 predicted cDNA sequences, revealing 12 expressed functional *PHO1*-like genes in the soybean genome. No transcription was observed for *GmaPHO1; H11* on chromosome 2 and *GmaPHO1; H13* on chromosome 20 in Nannong1138-2.

**Table 1:** **Molecular characterization of *GmaPHO1 *genes in soybean**

**Gene**	**Locus name**	**Chr**^**a**^	**PL**^**b**^	**SL**^**c**^	**TMS**^**d**^
***GmaPHO1; H1***	Glyma02g00640	02	763	PM, Nuc	7
***GmaPHO1; H2***	Glyma07g35520	07	801	PM, Nuc	8
***GmaPHO1; H3***	Glyma09g37000	09	756	PM, Nuc	6
***GmaPHO1; H4***	Glyma10g00720	10	764	PM, Nuc	5
***GmaPHO1; H5***	Glyma10g32670	10	771	PM, Nuc	7
***GmaPHO1; H6***	Glyma18g49680	18	773	PM, Nuc	6
***GmaPHO1; H7***	Glyma20g03960	20	784	PM, Nuc	5
***GmaPHO1; H8***	Glyma20g34930	20	771	PM, Nuc	7
***GmaPHO1; H9***	Glyma20g04130	20	792	PM, Nuc, ER	7
***GmaPHO1; H10***	Glyma20g04150	20	804	PM, Nuc, ER	9
***GmaPHO1; H11***	Glyma20g04160	20	817	PM, Nuc	8
***GmaPHO1; H12***	Glyma01g22990	01	804	PM, Nuc, ER	7
***GmaPHO1; H13***	Glyma02g12320	02	753	PM, ER	6
***GmaPHO1; H14***	Glyma02g14440	02	789	PM, Vac	6

PM, plasma membrane; Nuc, nuclear; Vac, vacuolar membrane; ER, endoplasmic reticulum. Chr^a^, Location of *GmaPHO1* genes on chromosomes. PL^b^ Peptide length (aa). SL^c^**,** Subcellular location of GmaPHO1 proteins. TMS^**d**^, Number of transmembrane segments.

### Organ-specific expression of *GmaPHO1* genes during soybean development

The expression of *GmaPHO1* genes using real-time RT-PCR approach provided clues to their functional divergence. Total RNAs used in this study was taken from roots, leaves, stems, flowers, and developing fruits of soybean cultivar Nannong1138-2.

**Figure 3:**
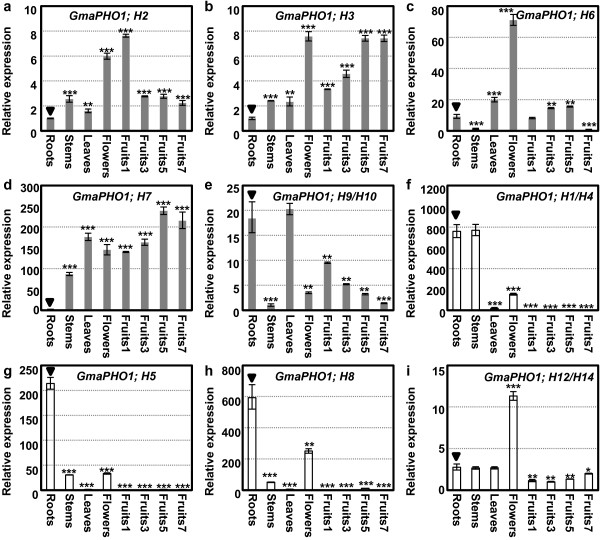
**Organ-specific expressions of *GmaPHO1 *genes during development.** (**a**-**e**) Organ-specific expressions of the Class I genes. (**f**-**i**) Organ-specific expressions of the Class II genes. The gene is indicated in each graph. Roots, stems, leaves, mature flowers and fruits (1-, 3-, 5- and 7-day fruits after pollination) are involved. *Actin* was used as an internal control. The experiments were performed based on three independent biological samples. Error bars=standard deviations. The star (*P < 0.05, **P < 0.01 and ***P < 0.001) represented the statistical significance of the gene expression variation in other tissues compared to that in roots indicated by triangles.

Two-tailed Student’s *t*-test of gene expression variation among these organs confirmed diverse expression patterns (see Additional file 12: Figure S5). Here, we report difference in gene expression in plant organs compared with that in roots. Under normal condition, most *GmaPHO1* genes were constitutively expressed in different tissues, but several genes had tissue-specific or preferential expression patterns (Figure 3). In Class Ι (Figure 3a-e, highlighted in grey column), *GmaPHO1; H2* and *GmaPHO1; H3* were found to be expressed in all organs examined; however, they were significantly abundant in flowers and fruits (*P*<0.001). *GmaPHO1; H2* was significantly down-regulated while *GmaPHO1; H3* was significantly up-regulated during fruit development (Figure 3a and b). *GmaPHO1; H6* was predominantly expressed in flowers and leaves (*P*<0.001) and was moderately expressed in roots and earlier fruit developments with low expression in stems and 7-day fruits after pollination (Figure 3c). The expression level of *GmaPHO1; H7* was found to be significantly higher in most organs and maintained a higher level during fruit development, but undetectable in roots (*P*<0.001) (Figure 3d). *GmaPHO1; H9/H10* were expressed in most tissues; however, their expression levels were found to be much higher in roots and leaves (*P*<0.01) and extremely lower in stems and 7-day fruits (*P*<0.001). Notably, these genes were observed to be gradually repressed during fruit development (Figure 3e). However, genes in Class II had limited organ expression patterns with clear difference between Class IIA and Class IIB (Figure 3f-i, highlighted in empty column). Genes in Class IIA were predominantly expressed in roots; however, they were expressed to a lesser degree in flowers and were not expressed in leaves and developing fruits (*P*<0.001). *GmaPHO1; H1/H4* had an equivalent expression in stems and roots, while *GmaPHO1; H5* and *GmaPHO1; H8* were less expressed in stems (Figure 3f-h). *GmaPHO1; H12/H14* in Class IIB were constitutively expressed, and predominantly so in mature flowers (*P*<0.001) (Figure 3i). These findings suggest that *GmaPHO1* genes may exert certain roles in soybean development with lineage-specific divergent patterns.

### Gene expression in response to various stresses in soybean

**Figure 4:**
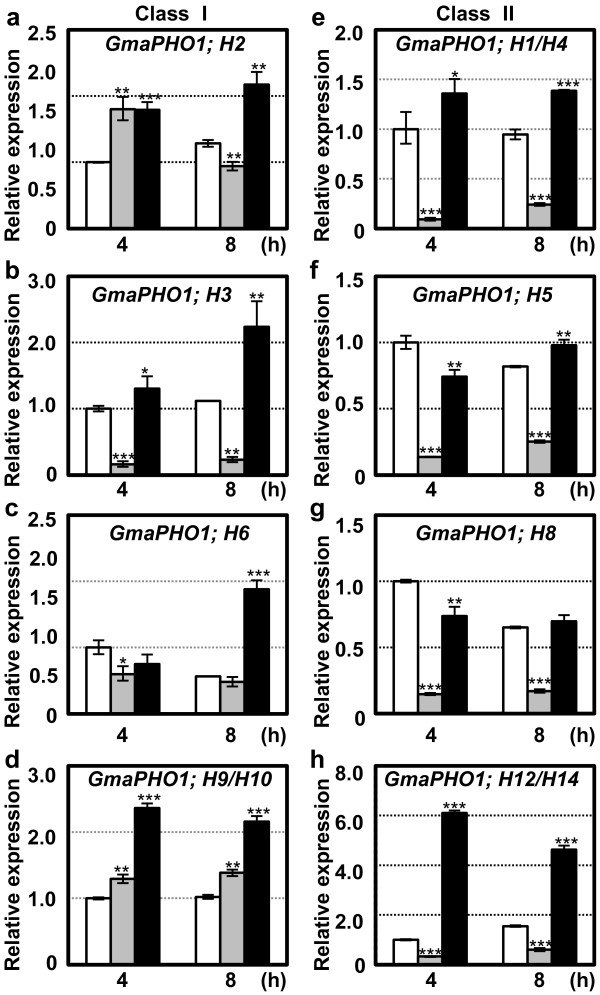
**Expression of *GmaPHO1 *genes in response to salt and drought stresses.** (**a**-**d**) Root expression of the Class I gene in response to PEG-6000 (grey column) or NaCl (black column). (**e**-**h**) Root expression of the Class II gene in response to PEG-6000 (grey column) or NaCl (black column). Genes are indicated in each graph. Empty column represents the untreated control. The 3-week old seedlings of ‘Nannong1138-2’ were treated with for 4 h and 8 h. *Actin* was used as an internal control. The experiments were performed based on three independent biological samples. Error bars=standard deviations. Star indicates the significance of gene expression variation under each treatment in comparison to the untreated control (*P < 0.05, **P < 0.01 and ***P < 0.001).

Examination of soybean *PHO1* genes transcription in roots challenged with osmotic, salt stress, and Pi stresses in the 3-week old seedlings (see Methods) identified genes that respond to abiotic stresses. Total RNA from roots was subjected to real-time RT-PCR for *GmaPHO1*genes (except for *GmaPHO1; H7* due to its silencing in roots; Figure 3d). Relative to untreated controls (Figure 4, empty column), Class I genes had different responses to osmotic stress (Figure 4a-d, highlighted in grey column), while the genes in Class II responded similarly to osmotic stress (Figure 4e-h, highlighted in grey column). In Class I, *GmaPHO1; H3* was rapidly and constantly repressed (*P*<0.01) (Figure 4b), and *GmaPHO1; H9/H10* were induced by osmotic stresses (*P*<0.01) (Figure 4d). The genes *GmaPHO1; H2* or *GmaPHO1; H6* were slightly influenced by osmotic treatments (*P*<0.05) (Figure 4a and c). Class IIA genes were repressed by osmotic stresses (*P*<0.001) (Figure 4e-h, highlighted in grey column). Under salt stresses, all Class Ι genes were induced to express (*P*<0.05) (Figure 4a-d, highlighted in black column). In Class IIA, genes were moderately changed under the influence of salt stress (Figure 4e-g). In contrast, *GmaPHO1; H12/H14* in Class IIB were strongly induced under salt stress conditions (*P*<0.001); their expression increased more than 6 times compared with controls (Figure 4h). The two types of Pi stress treatments altered *GmPHO1* gene transcription significantly at most treatment time-points (see Additional file 13: Figure S6); however, each gene had complex but similar pattern under the Pi stress treatments, suggesting that *GmPHO1* genes have an complex response to Pi alteration around the roots. Thus, these genes have different roles in response to salt, osmotic and Pi stresses.

## Discussion

Limited functional characterization of the *PHO1* gene family suggests it has important roles in plant developmental and physiological processes [1,3-7,12,13,15-17]. Here we report our investigations into the phylogeny and structural evolution of *PHO1*-like genes in plants and share data about their functional divergence. Our data were confirmed with comprehensive expression analyses of these genes in soybean.

### Evolution of the *PHO1* gene family during evolution of plants

**Figure 5:**
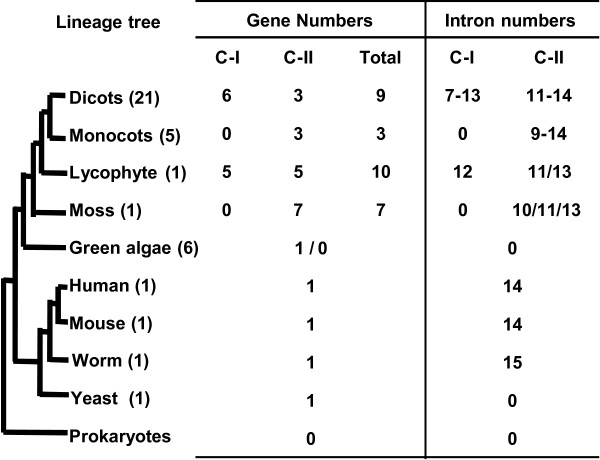
**Evolutionary patterns of the *PHO1 *gene family.** Variations in both gene copy and introns of the *PHO1* genes in the major lineages of organisms are summarized. Liverworts and gymnosperms are not included as their e whole genome sequences were not available. The number of the species with whole genome sequenced is given in the parenthesis behind of each lineage. Gene copy number of the lineages with multiple species is given as the mean per species. Class I and Class II, are abbreviated as C-I and C-II respectively.

Our results revealed that *PHO1* homologs were prevalent in eukaryotes only. Unlike previous reports [9,10,28], we identified two *PHO1*-like cDNAs in two of the six released genomes of chlorophyta, indicating that the gene is not required for all unicellular green algae. However, *PHO1*-like genes were expanded tremendously in multicellular plants (Figure 5). Phylogenetic reconstruction with an 87% bootstrap value revealed that these genes formed two major classes in land plants (Class I, II), indicating that the *PHO1*-like genes from all land plants share a common ancestor; the two classes are sister groups. The topology of the *PHO1* gene family of land plants might have originated through several duplications, followed by gene loss in some descendants. Genes in Class I, occupying 58.8% (130/221) of the total identified genes, were from 21 dicots and one lycophyte, while 91 members from land plants were present in Class II (Figure 5). One duplication event led to the division of Class II into Class IIA and Class IIB of the genes in seed plants, while multiple duplication events including tandem duplications in dicots that specifically occurred in Class I led to its expansion. These data indicated that lineage-specific expansion and divergence events of the *PHO1*-like genes occurred in seed plants.

Gene duplication is a permanent and continual factor contributing to the complexity of genetic material [23,31-33]. Gene copy variation, a result of gene duplication and gene loss, was prominent in the *PHO1* gene family. The gene family also evolved in yeast, animals and green algae with a single copy, while copy numbers of these genes in land plants varied between 2 and 23. The variable copy numbers are not consistent with the genome sizes of different species, evidence for which was obtained from an early vascular plant with a small genome (100 Mb). There are 10 copies of *PHO1* genes in *S.moellendorffii*, whereas in *Z.mays* (2400 Mb) only 2 copies survived. However, gene family expansion slightly correlates to the whole-genome duplication and triplication events which occurred among Viridiplantae [23-25], indicating an important role of gene losses during *PHO1* gene family evolution. Monocots were found to possess lesser *PHO1*-like genes compared to dicots and basal land plants, like *P.patens* and *S.moellendorffii*. There are 185 members in 21 dicots compared to 13 members in 5 monocots (Figure 5); thus, gene loss events are key to monocots evolution. These results imply that the expansion of the *PHO1* gene family may be associated with the origin of diversification of land plants.

A genome-wide study compared orthologous groups of genes in 4 model genomes (*Arabidopsis*, poplar, rice, and *P. patens*) and revealed that genes related to intracellular components, regulation of metabolism, hormone metabolism, transcriptional regulation, cell communication, and responses to hormone stimuli are more likely to have undergone non-tandem duplication. In contrast, genes involved in stress responses had an increased probability of being retained in a single-lineage fashion after tandem duplication, suggesting that tandem duplicates are probably important for adaptations to rapidly changing environments [33]. Interestingly, we found that almost 50% of Class ΙA genes (55/125) underwent tandem duplication. However, found no tandem repeats in Class II. *PHO1*-like genes in Class II have been functionally characterized in *Arabidopsis* and rice [1,5,8-10] and are known to play major roles in root-to-shoot transfer of phosphate, while Class Ι genes are important in biotic and/or abiotic stress responses [15-17]. Tandem gene arrangements and copy number variation of *PHO1* Class ΙA genes may arise from a response to different environmental selections during evolution. This assumption was further supported by our expression analyses in soybeans.

The majority of conserved introns remain unchanged over time [34,36]. Thus, intron/exon changes could provide clues on evolutionary relationships [37,38]. The intronless *PHO1* genes originated from a eukaryotic ancestor, evidence for which is provided by the structure of unicellular yeast and green algae (Figure 5). Once multicellular organisms (mammals and land plants) appeared, *PHO1* genes became more complicated. Introns can probably be gradually acquired, or obtained once during the evolution of the multicellular organisms from the unicellular ancestors. Our gene structure reconstruction and exon-intron analysis with all available sequences revealed that the gene structure with 13 introns might be an original organization in land plants, and that *PHO1* genes might have evolved along with multicellular organisms (Figure 5). We therefore proposed that the intron loss is a major event in the evolution of *PHO1* genes of land plants. Most Class I members had fewer introns than their ancestors, due to intron loss accompanying the exon-fusion event. This event occurred less frequently in Class IIA and Class IIB members, therefore, most of them contained 13 or 14 introns and the 14-intron structure arose from an additional novel intron gain (Figure 5; see Additional file 6: Figure S2), which only occurred in Class IIA. Also, intron losses occurred independently, resulting in 12–14 introns. Consistent with the previous observation that intron phase change is rare [38], intron phases of adjacent exons were found to be almost conserved in *PHO1* genes in different classes.

Furthermore, we found that 119 of 130 genes lost their first intron in Class I. Plant studies offer evidence of intron effects on gene expression [39-42]. The first intron of *Arabidopsis* histone H3 gene, *Petunia* actin-depolymerizing factor 1 (*PhADF1*) and maize *shrunken-1* (*Sh1*) can alter gene expression levels [43-45]. Introns are also involved in the regulation of spatial and temporal gene expression patterns, such as photosystem I subunit II (*PsaD*), *AGAMOUS* (*AG*) and *flowering locus C* (*FLC*) [46-48]. In the dicot-specific Class IA, almost all genes lost their first introns. Indeed, the *Arabidopsis* Class I *PHO1* genes have a boarder expression domain than Class II genes [7]. Our data support these finding: expression domains of the soybean genes in Class I are more diverse than those in Class II.

### Functional diversification of the *PHO1* gene family in soybean

We surveyed the genome draft of soybean Williams 82 and predicted 14 *PHO1*-like genes. They were found to be either tandemly or segmentally located on the chromosomes. We characterized the cDNA sequences from the Nannong1138-2 cultivars to validate 12 of the predicted sequences. These genes on the phylogenetic tree were classified into the Class I and Class II, suggesting their functional divergence in developmental processes and in response to abiotic stresses.

Transcripts of the predicated two genes (*GmaPHO1; H11* and *GmaPHO1; H13*) in Class IA were not detected in Nannong 1138–2, indicating that either these genes were silenced or had extremely low levels. The expression profiles of soybean *PHO1-*like genes provided clues to their functional relevance and divergence. We found that the genes in the two classes were expressed with different tissue-specific patterns, while the genes in the same class showed a similar expression pattern. The genes in Class I had a broader expression domain than those in Class II and they were developmentally regulated during fruit development in soybean. These results are corroborated by previous studies on *AthPHO1; H4* in Class I that plays a role in the response of hypocotyls to blue light [11], as well as seed size and flowering [12-14]. The genes in Class IIB were expressed in all tissues examined, while the genes in Class IIA seemed to be restricted to roots, stems and flowers. These findings, therefore, support a lineage-specific functional differentiation related to soybean plant and fruit development.

The phylogenetic analysis showed that *GmaPHO1; H1*, *GmaPHO1; H4*, *GmaPHO1; H5* and *GmaPHO1; H8* are grouped with *AthPHO1* and *OsaPHO1; H2* that play important roles in Pi transference [7,9], hinting that these genes might have related functions. These genes indeed showed a similar tissue-specific expression pattern with little variation. Most *GmaPHO1* genes were expressed in roots and responded to Pi deficient or sufficient treatments in complex manners. Surprisingly, each gene in the different Pi stresses had a similar responding mode in most treated-time points. This is not well understood yet. Nonetheless, our observations suggest that they might function in Pi sensing or transporting in soybean as their homologs *AthPHO1* in *Arabidopsis* and *OsaPHO1; H2* in rice [1,7,9].

*AthPHO1; H10* in *Arabidopsis* is activated by a diversity of stimuli including salinity, osmotic, pathogens and even by wounding [15], thus suggesting that some *PHO1-*like genes might be involved in these physiological processes. *GmaPHO1* genes exhibited varied expression patterns under abiotic stresses. The genes, *GmaPHO1; H3* in Class IA, *GmaPHO1; H1/H4*, *GmaPHO1; H5*, *GmaPHO1; H8* and *GmaPHO1; H12/H14* in Class IIA were rapidly and constantly inhibited by osmotic stress, while all genes in Class IA and *GmaPHO1; H12/H14* in Class IIB were found to be strongly induced by salt stress, suggesting that they are likely associated with the processes that respond to these stresses. Inspection of chromosomal localization of *PHO1* gens in soybean and other species genomes suggests that almost 50% of the genes in Class IA are arranged tandemly on the chromosomes. Our study combined with the evidences from *Arabidopsis* supports the deduction that tandem duplication and variation in copy number of *PHO1* gene in Class ΙA might result from a response to different environmental selection during evolution. During evolution, modulating gene expression and increasing gene numbers possibly allows the different *PHO1*genes to act synergistically to promote plant development and to reinforce tolerance to environment stresses. These genes can greatly contribute to plant adaptation and survival in adverse conditions through offsetting the effects of mutations.

## Conclusions

Using comparative genomic and phylogenetic analyses we identified two major Classes (I and II) of the *PHO1* gene family in land plants. Class I genes were expanded in dicotyledonous plants and *Selaginella moellendorffii*, while Class II genes were found in all land plants. Class IA subfamily gene expansion in dicots and the loss of the subfamily genes in monocots were also documented. Intron loss was found to be an active force in the evolution of gene structure since the origin of a complicated structure (14-*EXONS*) in multicellular plants. The functional divergences among *PHO1 *genes in soybean suggested that significant subclass-specific functional evolution took place after their phylogenetic diversification. Our results suggest that the expansion and sequence variation accompanying dramatic functional diversifications of the plant *PHO1* gene family are associated with the origin of diversification of land plants. Our findings provided valuable information and new insights into the molecular and functional evolutionary pattern of the plant gene family during evolution of plants. Evaluating the function of this gene family could provide valuable insights into their involvement in metabolic and biochemical processes.

## Methods

### Identification and physical locations of *PHO1* genes in soybean

*Arabidopsis PHO1 *orthologs were used to search the National Center for Biotechnology Information (NCBI; http://www.ncbi.nlm.nih.gov) and Phytozome (http://www.phytozome.net) databases. The primers used for cloning *PHO1s* were designed from the 5′ends and 3′ ends of putative coding regions according to their linear alignment with *Arabidopsis* genes. For those cDNA fragments lacking 5′ and/or 3′ ends of the coding regions, 5′-RACE and/or 3′-RACE was performed to obtain the missing regions (Roche, Mannheim, Germany). *PHO1*-like cDNAs were amplified by PCR from a combination of cDNAs from different soybean tissues using gene specific primers as described in Additional file 11: Table S7. Genes were mapped on chromosomes by identifying their chromosomal position provided in the Phytozome database. Protein subcellular localization was predicted using PSORT software (http://wolfpsort.org).

### Phylogenetic tree constructions

To analyze the sequence features of the 223 typical identified *PHO1* genes in plants, a multiple sequence alignment (MSA) was carried by the GUIDANCE web-server using the MSA algorithm MAFFT (http://guidance.tau.ac.il/index.html) [49], and eliminated the poorly aligned positions were further using the G-blocks server (http://molevol.cmima.csic.es/castresana/Gblocks_server.html) [50,51]. ProtTest version 2.4 [52] was used to conclude that JTT model was the best-fit model for amino acid evolution according to both Akaike Information Criterion (AIC) and Bayesian Information Criterion (BIC). To obtain optimized alignment, the deduced amino acid sequences were adjusted manually using BioEdit (v7.0.5) [53]. The ML phylogenetic tree was constructed using PhyML (v3.0) [54] under the JTT amino acid substitution model, with 100 replicates of bootstrap analysis, estimated gamma distribution parameter, and optimized starting BIONJ tree. The tree was deposited in the TreeBASE (S14085 Matrix ID). ML trees generated using both WAG and LG models had a topology similar to the JTT model, and these are presented in Additional file 2: Figure S1. The phylogenetic tree was visualized using the TreeView1.6.6 tool with a 50% threshold of branch value [55]. Notung software (version 2.6) was used for tree reconciliation [56,57]. The species tree used to reconcile the gene tree was based on taxonomic information from the NCBI database. Correlations between gene copy numbers and genome size as well as whole-genome duplication events timing was estimated using the Pearson correlation with a two tailed significance test. Statistical analysis was performed in SPSS 15.0 for Windows.

### Gene structure analyses

For intron/exon structure analysis, the DNA and cDNA sequences corresponding to each predicted gene from the Phytozome database annotation were downloaded, and their intron distribution patterns and splicing phases were analyzed manually using BioEdit (v7.0.5). Intron phases were determined as follows: splicing occurring after the third nucleotide of the codon as phase 0, splicing occurring after the first nucleotide of the codon as phase 1 and splicing occurring after the second nucleotide as phase 2. To trace the plant *PHO1* gene structures evolution, we conducted the character state reconstruction using Mesquite version 2.72 (http://mesquiteproject.org) [58]. The structure of the *PHO1* genes possesses 19 character states. They are a)14-EXON type , b) 1^st^-Intron loss type c) 2^nd^-Intron loss type, d) 3^rd^-Inron absent type, e) 4^th^-Intron absent type, f) 5^th^-Intron absent type, g) 6^th^-Intron absent type, h) 7^th^-Intron absent type, i) 8^th^-Intron absent type, j) 9^th^-Intron absent type, k) 10^th^-Inrton absent type, l) 11^th^-Intron absent type, m) 12^th^-Intron absent type, n) 13^th^-Intron absent type, o) Intronless type, p) 1^st^-Intron insertion type, q) Animal-14-Intron type, r) Animal-15-Intron type, and s) unknown type. A total of 227 matrices reflecting the gene structures were built, and the topology reflecting the phylogenetic tree of *PHO1* genes was used as the input tree. Reconstruction of the ancestral state was performed using MP criterion. The unordered model in which all state changes are treated equally was applied for the parsimony analyses.

### Plant growth and stress treatments

Soybean (*Glycine max* L.) Nannong1138-2 cultivars were grown in vermiculite in a greenhouse at 22±2°C with a 16/10 h (light/dark) photoperiod. Roots, leaves, and stems were harvested from 3-week-old seedlings. Mature flowers and the fruits of 1-, 3-, 5-, and 7-days after fertilization were sampled in the greenhouse. For stress treatments, 3-week-old seedlings were grown in a 0.5×Hoagland solution (pH5.8). Drought stress was mimicked by submerging seedlings in Hoagland solution containing 15% (w/v) PEG 6000 for 4 and 8 h. The seedlings were subjected to salt stress by growing them in Hoagland solution containing 150 mM NaCl for 4 and 8 h. We designed 2 experiments for Pi stress: (1) first subjecting the seedlings to low Pi stress (LP, 0 mM phosphorus) for 24 h, and then high Pi stress (HP, 5 mM phosphorus) for 24 h. The second treatment approach was to reverse the stresses, presenting HP first for 24 h and then LP for 24 h. Untreated seedlings in Hoagland solution were used as controls for all samples. Roots were harvested at the indicated times. Collected plant materials were flash-frozen in liquid nitrogen and then stored at −80°C for RNA isolation.

### Real-time RT-PCR analysis

Total RNAs was extracted with the Trizol Reagent (Invitrogen, Carlsbad, USA) and treated with the RQ1 RNase-free DNase (Promega, Madison, WI) to avoid DNA contamination. Then, 2.0 μg total RNA was used to synthesize cDNA with the oligo (dT)_18_ primer following the instructions of the M-MLV cDNA synthesis kit (Invitrogen, Carlsbad, CA). Real time RT-PCR analysis was performed on an Mx3000P QPCR system (Stratagene, Germany) using SYBR Premix Ex Taq (TaKaRa, Tokyo, Japan). Gene-specific primers are listed in Additional file 11: Table S7. Primers specificity was verified using the BLAST tool from the NCBI and a dissociation curve analysis was performed after the PCR reaction. The efficiency E value was calculated for all primer pairs individually by plotting the relationship between Ct value (threshold cycle) and log [cDNA]. PCRs were done using the following thermal cycle conditions: 95°C for 30 s, followed by 40 cycles of 95°C for 5 s and 60°C for 40 s. Controls without template were included for each primer pair and all reactions were repeated three times for 3 independent biological samples. Relative gene expression was analyzed using the cycle threshold (Ct) 2^-ΔΔ^Ct method [59] and normalized using the housekeeping gene *actin*. Two-tailed Student’s *t*-test was used to determine the statistical significance of gene expression variation between different tissues and in response to stresses (*P < 0.05, **P < 0.01 and ***P < 0.001). Statistical analysis was performed in SPSS 15.0 for Windows.

## Abbreviations

ML: Maximum-likelihood; PHO1: *PHOSPHATE1*; RT-PCR: Reverse transcription-polymerase chain reaction amplification; RACE: Rapid amplification of cDNA ends.

## Competing interests

The authors declare that they have no competing interests.

## Authors’ contributions

CYH designed the study and conceived the experiments. LLH characterized the sequences and carried out most of the experiments. YW participated expression analyses. LLH and MZ performed evolutionary analyses. LLH and CYH analyzed data. JYG and CYH coordinated the work. LLH and CYH drafted the manuscript. All authors read and approved the final manuscript.

## Supplementary Material

Additional file 1: Table S1 Information of the *PHO1* gene family used in the present work. Click here for file

Additional file 2: Figure S1 ML trees generated using both WAG and LG models. (**a**) ML tree generated under LG model. (**b**) MLtree generatedunder WAG model. Bootstrap values (>50%) for this tree are shown on each branch. The different classes are marked with different colored backgrounds. The genes from the basal land plants are indicated in pink; dicot in red, monocots in blue and gymnosperm in green, respectively. Click here for file

Additional file 3: Table S2 Copy numbers of *PHO1* genes in different classes in 30 land plant species. Click here for file

Additional file 4: Table S3 The presence of tandem duplications in Class IA. Click here for file

Additional file 5: Table S4 Percent of members with different numbers of introns in each class. Click here for file

Additional file 6: Figure S2 Evolution of intron gain and loss events of the plant *PHO1* gene family. ML tree of the plant *PHO1* genes is identical to **Figure1**. Ancestral state reconstruction of *PHO1* gene structure was conducted by Mesquite. Color scale depicts 19 different intron states of gene structures. Pie charts linked with the internal branches represent probability of ancestral states. Solid (one color) node indicates that the particular state is significantly better than all other possible states. Gene structures are based on the predicted sequences from the Phytozome database. The intron numbers corresponding to the assumed ancestral *PHO1* genes are shown at the top. Numbers in boxes represent intron phases 0, 1 or 2, respectively. Black boxes indicate conserved introns, white boxes indicate lost introns, and gray boxes indicate inserted introns. Click here for file

Additional file 7: Figure S3 Chromosomal location of *GmaPHO1* genes. The horizontal line represents the chromosome and the vertical lines repre**s**ent the *GmaPHO1* genes. Arrows indicate the transcriptional directions of the corresponding genes. *H1-H14* are abbreviated for *GmaPHO1; H1-H14* genes. Click here for file

Additional file 8: Table S5 Variation in exon number and length of *GmaPHO1* genes. Click here for file

Additional file 9: Figure S4 Multiple sequence alignment of PHO1 proteins in soybean, *Arabidopsis* and rice. The characteristic domains of the PHO1 proteins are highlighted in red (SPX, Pfam PF03105) and pink (EXS, Pfam PF03124) lines, respectively. Click here for file

Additional file 10: Table S6 Pairwise comparison of the GmaPHO1 protein family. Click here for file

Additional file 11: Table S7 Primer sequences of *GmaPHO1* genes used in the present work. Click here for file

Additional files 12: Figure S5 The reciprocal significance of gene expression variation among tissues. (**a-e**) Significance of organ-specific expressions the Class I genes. (**f-i**) Significance of organ-specific expressions of the Class II genes. Click here for file

Additional files 13: Figure S6 Expression of *GmaPHO1* genes under Pi deficient or sufficient treatments. (**a-d**) Root expression of the Class I genes in response to Pi stresses. (**e-h**) Root expression of the Class II genes in response to Pi stresses. The gene is indicated in each graph. Light grey lines represent the untreated controls; dark gray lines represent the treatment of LP-HP; black lines represent the treatment of HP-LP (see Methods). *Actin* was used as an internal control. The experiments were performed based on three independent biological samples. Error bars=standard deviations. Asterisks indicate significance of gene expression variation under each treatment in comparison to the untreated control (*P < 0.05, **P < 0.01 and ***P < 0.001). Click here for file
