# Supplementary material for: Phylogeny, structural evolution and functional diversification of the plant PHOSPHATE1 gene family: a focus on Glycine max
Source: BMC Evol Biol. 2013 May 24;13:103. doi: 10.1186/1471-2148-13-103 (PMC3680083; doi:10.1186/1471-2148-13-103)
Supplement: Additional file 2: Figure S1 — ML trees generated using both WAG and LG models. (a) ML tree generated under LG model. (b) MLtree generatedunder WAG model. Bootstrap values (>50%) for this tree are shown on each branch. The different classes are marked with different colored backgrounds. The genes from the basal land plants are indicated in pink; dicot in red, monocots in blue and gymnosperm in green, respectively. [file 1471-2148-13-103-S2.pptx]

## Slide 1
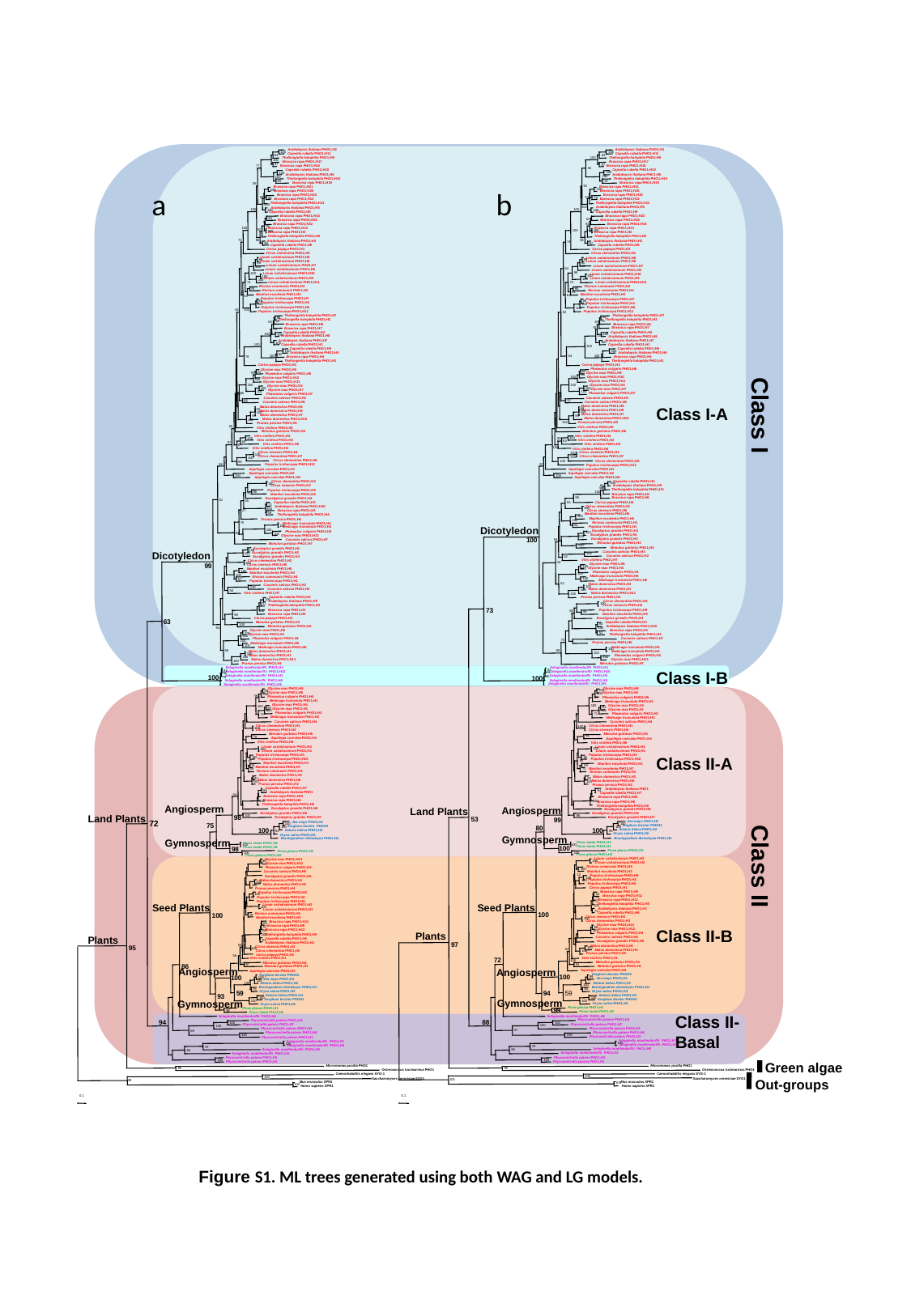

Arabidopsis thaliana PHO1;H2
100
Capsella rubella PHO1;H11
64
Thellungiella halophila PHO1;H9
100
Brassica rapa PHO1;H17
97
Brassica rapa PHO1;H16
77
Capsella rubella PHO1;H10
100
Arabidopsis thaliana PHO1;H6
100
Thellungiella halophila PHO1;H10
85
Brassica rapa PHO1;H19
58
Brassica rapa PHO1;H21
100
Brassica rapa PHO1;H20
Brassica rapa PHO1;H18
59
86
Brassica rapa PHO1;H13
Thellungiella halophila PHO1;H11
100
Arabidopsis thaliana PHO1;H5
100
100
Capsella rubella PHO1;H9
Brassica rapa PHO1;H15
50
Brassica rapa PHO1;H23
Brassica rapa PHO1;H22
93
Brassica rapa PHO1;H14
100
90
100
Brassica rapa PHO1;H2
Thellungiella halophila PHO1;H8
98
70
Arabidopsis thaliana PHO1;H3
99
Capsella rubella PHO1;H8
Carica papaya PHO1;H3
Citrus clementina PHO1;H5
Linum usitatissimum PHO1;H8
99
Linum usitatissimum PHO1;H6
66
Linum usitatissimum PHO1;H7
100
Linum usitatissimum PHO1;H5
100
Linum usitatissimum PHO1;H10
100
Linum usitatissimum PHO1;H9
100
Linum usitatissimum PHO1;H11
74
Ricinus communis PHO1;H3
91
Ricinus communis PHO1;H2
Manihot esculenta PHO1;H3
63
Populus trichocarpa PHO1;H7
99
Populus trichocarpa PHO1;H4
100
Populus trichocarpa PHO1;H6
100
65
Populus trichocarpa PHO1;H11
Thellungiella halophila PHO1;H7
88
Thellungiella halophila PHO1;H2
69
83
Brassica rapa PHO1;H5
Brassica rapa PHO1;H7
70
Capsella rubella PHO1;H2
100
100
Arabidopsis thaliana PHO1;H8
Arabidopsis thaliana PHO1;H7
100
Capsella rubella PHO1;H1
100
Capsella rubella PHO1;H5
100
Arabidopsis thaliana PHO1;H4
62
100
78
Brassica rapa PHO1;H4
Thellungiella halophila PHO1;H1
Carica papaya PHO1;H2
Glycine max PHO1;H9
67
79
Phaseolus vulgaris PHO1;H6
Glycine max PHO1;H10
100
Glycine max PHO1;H11
100
Glycine max PHO1;H2
100
Glycine max PHO1;H7
100
55
20
Phaseolus vulgaris PHO1;H7
Cucumis sativus PHO1;H1
86
Cucumis sativus PHO1;H6
Malus domestica PHO1;H8
50
Malus domestica PHO1;H9
100
Malus domestica PHO1;H7
99
Malus domestica PHO1;H10
100
Prunus persica PHO1;H3
55
Vitis vinifera PHO1;H6
Mimulus guttatus PHO1;H6
Vitis vinifera PHO1;H3
100
Vitis vinifera PHO1;H2
85
97
Vitis vinifera PHO1;H5
87
Vitis vinifera PHO1;H4
Citrus sinensis PHO1;H1
100
Citrus clementina PHO1;H7
100
Citrus clementina PHO1;H6
99
98
Populus trichocarpa PHO1;H12
Aquilegia coerulea PHO1;H1
Aquilegia coerulea PHO1;H2
100
Aquilegia coerulea PHO1;H5
Citrus clementina PHO1;H4
100
Citrus sinensis PHO1;H2
Populus trichocarpa PHO1;H9
98
75
Manihot esculenta PHO1;H5
Eucalyptus grandis PHO1;H4
50
55
Capsella rubella PHO1;H3
72
Arabidopsis thaliana PHO1;H10
100
27
Brassica rapa PHO1;H3
59
Thellungiella halophila PHO1;H4
Prunus persica PHO1;H5
78
Medicago truncatula PHO1;H4
100
Medicago truncatula PHO1;H3
100
Phaseolus vulgaris PHO1;H2
98
100
Glycine max PHO1;H13
Cucumis sativus PHO1;H7
Mimulus guttatus PHO1;H7
Eucalyptus grandis PHO1;H1
100
Eucalyptus grandis PHO1;H2
100
Eucalyptus grandis PHO1;H3
Citrus clementina PHO1;H2
100
99
Citrus sinensis PHO1;H5
Manihot esculenta PHO1;H6
100
Manihot esculenta PHO1;H4
100
Ricinus communis PHO1;H1
57
Populus trichocarpa PHO1;H1
Cucumis sativus PHO1;H3
100
Cucumis sativus PHO1;H2
56
Vitis vinifera PHO1;H7
Capsella rubella PHO1;H4
100
Arabidopsis thaliana PHO1;H9
51
Thellungiella halophila PHO1;H3
Brassica rapa PHO1;H1
100
Brassica rapa PHO1;H6
99
Carica papaya PHO1;H4
63
Mimulus guttatus PHO1;H3
100
Mimulus guttatus PHO1;H2
94
Glycine max PHO1;H6
100
Glycine max PHO1;H3
100
Phaseolus vulgaris PHO1;H1
100
Medicago truncatula PHO1;H5
100
Medicago truncatula PHO1;H6
68
Malus domestica PHO1;H2
100
Malus domestica PHO1;H1
100
Malus domestica PHO1;H11
100
Prunus persica PHO1;H1
Selaginella moellendorffii PHO1;H4
81
Selaginella moellendorffii PHO1;H10
95
100
Selaginella moellendorffii PHO1;H3
79
Selaginella moellendorffii PHO1;H8
Selaginella moellendorffii PHO1;H5
Glycine max PHO1;H8
99
Glycine max PHO1;H5
100
98
Phaseolus vulgaris PHO1;H5
Medicago truncatula PHO1;H1
Glycine max PHO1;H4
100
84
Glycine max PHO1;H1
100
Phaseolus vulgaris PHO1;H3
72
Medicago truncatula PHO1;H2
1
Cucumis sativus PHO1;H4
Citrus clementina PHO1;H1
100
Citrus sinensis PHO1;H4
Mimulus guttatus PHO1;H5
78
Aquilegia coerulea PHO1;H4
Vitis vinifera PHO1;H8
Linum usitatissimum PHO1;H4
100
Linum usitatissimum PHO1;H1
Populus trichocarpa PHO1;H3
100
Populus trichocarpa PHO1;H10
70
Manihot esculenta PHO1;H1
51
Manihot esculenta PHO1;H7
56
Ricinus communis PHO1;H4
Malus domestica PHO1;H3
81
Malus domestica PHO1;H6
100
Prunus persica PHO1;H2
Capsella rubella PHO1;H7
86
Arabidopsis thaliana PHO1
54
Brassica rapa PHO1;H10
Brassica rapa PHO1;H8
100
Thellungiella halophila PHO1;H6
Eucalyptus grandis PHO1;H8
Eucalyptus grandis PHO1;H6
98
100
Eucalyptus grandis PHO1;H7
72
Zea mays PHO1;H2
75
100
Sorghum bicolor PHOH2
100
100
Setaria italica PHO1;H2
91
Oryza sativa PHO1;H2
Brachypodium distachyon PHO1;H2
Pinus taeda PHO1;H2
100
Pinus taeda PHO1;H1
98
Picea glauca PHO1;H3
100
Picea glauca PHO1;H2
Glycine max PHO1;H14
96
Glycine max PHO1;H12
100
Phaseolus vulgaris PHO1;H4
Cucumis sativus PHO1;H5
Eucalyptus grandis PHO1;H5
Malus domestica PHO1;H4
100
Malus domestica PHO1;H5
100
Prunus persica PHO1;H4
Populus trichocarpa PHO1;H8
100
Populus trichocarpa PHO1;H2
99
Populus trichocarpa PHO1;H5
Linum usitatissimum PHO1;H2
100
Linum usitatissimum PHO1;H3
100
Ricinus communis PHO1;H5
97
Manihot esculenta PHO1;H2
Brassica rapa PHO1;H11
59
Brassica rapa PHO1;H9
82
Brassica rapa PHO1;H12
80
Thellungiella halophila PHO1;H5
100
Capsella rubella PHO1;H6
81
Arabidopsis thaliana PHO1;H1
58
95
Citrus sinensis PHO1;H3
100
Citrus clementina PHO1;H3
Carica papaya PHO1;H1
56
Vitis vinifera PHO1;H1
Mimulus guttatus PHO1;H4
100
87
86
Mimulus guttatus PHO1;H1
Aquilegia coerulea PHO1;H3
Sorghum bicolor PHOH3
100
100
Zea mays PHO1;H1
100
100
Setaria italica PHO1;H3
Brachypodium distachyon PHO1;H1
75
59
Oryza sativa PHO1;H3
93
Setaria italica PHO1;H1
100
Sorghum bicolor PHOH1
100
Oryza sativa PHO1;H1
Picea glauca PHO1;H1
98
Pinus taeda PHO1;H3
Selaginella moellendorffii PHO1;H6
94
Physcomitrella patens PHO1;H4
100
Physcomitrella patens PHO1;H7
100
Physcomitrella patens PHO1;H1
69
Physcomitrella patens PHO1;H6
100
Physcomitrella patens PHO1;H3
Selaginella moellendorffii PHO1;H7
100
Selaginella moellendorffii PHO1;H2
97
Selaginella moellendorffii PHO1;H9
85
Selaginella moellendorffii PHO1;H1
Physcomitrella patens PHO1;H5
100
Physcomitrella patens PHO1;H2
Micromonas pusilla PHO1
88
Ostreococcus lucimarinus PHO1
Caenorhabditis elegans SYG-1
100
Saccharomyces cerevisiae SYG1
99
Mus musculus XPR1
100
Homo sapiens XPR1
0.1
Arabidopsis thaliana PHO1;H2
100
Capsella rubella PHO1;H11
63
Thellungiella halophila PHO1;H9
100
Brassica rapa PHO1;H17
97
Brassica rapa PHO1;H16
84
Capsella rubella PHO1;H10
100
Arabidopsis thaliana PHO1;H6
100
Thellungiella halophila PHO1;H10
87
Brassica rapa PHO1;H19
50
Brassica rapa PHO1;H21
100
Brassica rapa PHO1;H20
Brassica rapa PHO1;H18
54
79
Brassica rapa PHO1;H13
Thellungiella halophila PHO1;H11
100
Arabidopsis thaliana PHO1;H5
100
100
Capsella rubella PHO1;H9
Brassica rapa PHO1;H23
52
Brassica rapa PHO1;H22
91
Brassica rapa PHO1;H15
Brassica rapa PHO1;H14
100
100
91
Brassica rapa PHO1;H2
Thellungiella halophila PHO1;H8
98
Arabidopsis thaliana PHO1;H3
74
97
Capsella rubella PHO1;H8
Carica papaya PHO1;H3
Citrus clementina PHO1;H5
Linum usitatissimum PHO1;H8
99
Linum usitatissimum PHO1;H6
60
Linum usitatissimum PHO1;H7
100
54
Linum usitatissimum PHO1;H5
98
Linum usitatissimum PHO1;H10
100
Linum usitatissimum PHO1;H9
100
70
Linum usitatissimum PHO1;H11
Ricinus communis PHO1;H3
90
Ricinus communis PHO1;H2
51
Manihot esculenta PHO1;H3
63
Populus trichocarpa PHO1;H7
98
Populus trichocarpa PHO1;H4
100
Populus trichocarpa PHO1;H6
100
Populus trichocarpa PHO1;H11
62
Thellungiella halophila PHO1;H7
84
Thellungiella halophila PHO1;H2
75
87
Brassica rapa PHO1;H5
61
Brassica rapa PHO1;H7
Capsella rubella PHO1;H2
100
100
Arabidopsis thaliana PHO1;H8
Arabidopsis thaliana PHO1;H7
100
Capsella rubella PHO1;H1
100
Capsella rubella PHO1;H5
100
Arabidopsis thaliana PHO1;H4
100
84
Brassica rapa PHO1;H4
Thellungiella halophila PHO1;H1
Carica papaya PHO1;H2
Phaseolus vulgaris PHO1;H6
60
Glycine max PHO1;H9
85
100
Glycine max PHO1;H10
Glycine max PHO1;H11
100
Glycine max PHO1;H2
100
Glycine max PHO1;H7
100
Phaseolus vulgaris PHO1;H7
Cucumis sativus PHO1;H1
77
Cucumis sativus PHO1;H6
Malus domestica PHO1;H8
58
Malus domestica PHO1;H9
100
Malus domestica PHO1;H7
99
Malus domestica PHO1;H10
100
Prunus persica PHO1;H3
Vitis vinifera PHO1;H6
Mimulus guttatus PHO1;H6
Vitis vinifera PHO1;H3
100
82
Vitis vinifera PHO1;H2
83
Vitis vinifera PHO1;H5
100
Vitis vinifera PHO1;H4
Citrus sinensis PHO1;H1
100
Citrus clementina PHO1;H7
100
Citrus clementina PHO1;H6
100
97
Populus trichocarpa PHO1;H12
Aquilegia coerulea PHO1;H1
Aquilegia coerulea PHO1;H2
100
Aquilegia coerulea PHO1;H5
Capsella rubella PHO1;H4
100
64
Arabidopsis thaliana PHO1;H9
56
Thellungiella halophila PHO1;H3
100
Brassica rapa PHO1;H1
99
Brassica rapa PHO1;H6
Carica papaya PHO1;H4
55
Citrus clementina PHO1;H2
100
Citrus sinensis PHO1;H5
Manihot esculenta PHO1;H6
100
Manihot esculenta PHO1;H4
100
Ricinus communis PHO1;H1
54
Populus trichocarpa PHO1;H1
Eucalyptus grandis PHO1;H1
100
Eucalyptus grandis PHO1;H2
100
100
Eucalyptus grandis PHO1;H3
56
Mimulus guttatus PHO1;H3
100
Mimulus guttatus PHO1;H2
Cucumis sativus PHO1;H3
100
Cucumis sativus PHO1;H2
52
Vitis vinifera PHO1;H7
92
Glycine max PHO1;H6
100
Glycine max PHO1;H3
100
Phaseolus vulgaris PHO1;H1
100
Medicago truncatula PHO1;H5
100
Medicago truncatula PHO1;H6
61
Malus domestica PHO1;H2
100
Malus domestica PHO1;H1
100
Malus domestica PHO1;H11
100
Prunus persica PHO1;H1
Citrus clementina PHO1;H4
100
Citrus sinensis PHO1;H2
73
Populus trichocarpa PHO1;H9
68
96
Manihot esculenta PHO1;H5
Eucalyptus grandis PHO1;H4
Capsella rubella PHO1;H3
84
Arabidopsis thaliana PHO1;H10
100
Brassica rapa PHO1;H3
51
Thellungiella halophila PHO1;H4
Cucumis sativus PHO1;H7
75
Prunus persica PHO1;H5
Medicago truncatula PHO1;H4
100
99
Medicago truncatula PHO1;H3
100
Phaseolus vulgaris PHO1;H2
100
Glycine max PHO1;H13
Mimulus guttatus PHO1;H7
Selaginella moellendorffii PHO1;H4
82
Selaginella moellendorffii PHO1;H10
96
Selaginella moellendorffii PHO1;H3
100
80
Selaginella moellendorffii PHO1;H8
Selaginella moellendorffii PHO1;H5
Glycine max PHO1;H8
100
Glycine max PHO1;H5
100
Phaseolus vulgaris PHO1;H5
97
Medicago truncatula PHO1;H1
Glycine max PHO1;H4
100
82
Glycine max PHO1;H1
100
Phaseolus vulgaris PHO1;H3
71
Medicago truncatula PHO1;H2
Cucumis sativus PHO1;H4
Citrus clementina PHO1;H1
100
Citrus sinensis PHO1;H4
Mimulus guttatus PHO1;H5
72
Aquilegia coerulea PHO1;H4
Vitis vinifera PHO1;H8
Linum usitatissimum PHO1;H4
100
Linum usitatissimum PHO1;H1
Populus trichocarpa PHO1;H3
98
Populus trichocarpa PHO1;H10
65
Manihot esculenta PHO1;H1
59
Manihot esculenta PHO1;H7
70
Ricinus communis PHO1;H4
Malus domestica PHO1;H3
83
Malus domestica PHO1;H6
100
Prunus persica PHO1;H2
Arabidopsis thaliana PHO1
94
Capsella rubella PHO1;H7
Brassica rapa PHO1;H10
100
Brassica rapa PHO1;H8
Thellungiella halophila PHO1;H6
Eucalyptus grandis PHO1;H8
Eucalyptus grandis PHO1;H6
96
Eucalyptus grandis PHO1;H7
53
99
Zea mays PHO1;H2
100
Sorghum bicolor PHOH2
80
100
100
Setaria italica PHO1;H2
95
Oryza sativa PHO1;H2
Brachypodium distachyon PHO1;H2
Pinus taeda PHO1;H2
100
100
Pinus taeda PHO1;H1
Picea glauca PHO1;H3
100
Picea glauca PHO1;H2
Linum usitatissimum PHO1;H2
100
Linum usitatissimum PHO1;H3
Ricinus communis PHO1;H5
100
Manihot esculenta PHO1;H2
10
Populus trichocarpa PHO1;H8
100
Populus trichocarpa PHO1;H2
100
Populus trichocarpa PHO1;H5
Carica papaya PHO1;H1
Brassica rapa PHO1;H9
52
Brassica rapa PHO1;H11
75
Brassica rapa PHO1;H12
82
Thellungiella halophila PHO1;H5
100
Arabidopsis thaliana PHO1;H1
71
100
Capsella rubella PHO1;H6
Citrus sinensis PHO1;H3
100
Citrus clementina PHO1;H3
Glycine max PHO1;H14
98
Glycine max PHO1;H12
100
Phaseolus vulgaris PHO1;H4
Cucumis sativus PHO1;H5
59
Eucalyptus grandis PHO1;H5
97
Malus domestica PHO1;H4
100
62
Malus domestica PHO1;H5
100
Prunus persica PHO1;H4
Vitis vinifera PHO1;H1
72
100
Mimulus guttatus PHO1;H4
91
Mimulus guttatus PHO1;H1
Aquilegia coerulea PHO1;H3
Sorghum bicolor PHOH3
100
99
Zea mays PHO1;H1
99
Setaria italica PHO1;H3
100
Brachypodium distachyon PHO1;H1
65
59
94
Oryza sativa PHO1;H3
Setaria italica PHO1;H1
100
Sorghum bicolor PHOH1
100
Oryza sativa PHO1;H1
Picea glauca PHO1;H1
99
Pinus taeda PHO1;H3
Selaginella moellendorffii PHO1;H6
Physcomitrella patens PHO1;H4
88
100
Physcomitrella patens PHO1;H7
100
Physcomitrella patens PHO1;H1
57
Physcomitrella patens PHO1;H6
100
Physcomitrella patens PHO1;H3
Selaginella moellendorffii PHO1;H7
100
Selaginella moellendorffii PHO1;H2
97
Selaginella moellendorffii PHO1;H9
86
Selaginella moellendorffii PHO1;H1
Physcomitrella patens PHO1;H5
100
Physcomitrella patens PHO1;H2
Micromonas pusilla PHO1
95
Ostreococcus lucimarinus PHO1
Caenorhabditis elegans SYG-1
100
100
100
Saccharomyces cerevisiae SYG1
Mus musculus XPR1
Homo sapiens XPR1
0.1
a
b
Class I-A
Class I
Dicotyledon
Dicotyledon
Class I-B
Class II-A
Angiosperm
Angiosperm
Land Plants
Land Plants
Gymnosperm
Gymnosperm
Class II
Seed Plants
Seed Plants
Plants
Class II-B
Plants
Angiosperm
Angiosperm
Gymnosperm
Gymnosperm
Class II-Basal
Green algae
Out-groups
Figure S1. ML trees generated using both WAG and LG models.
