# Supplementary material for: Phylogeny, structural evolution and functional diversification of the plant PHOSPHATE1 gene family: a focus on Glycine max
Source: BMC Evol Biol. 2013 May 24;13:103. doi: 10.1186/1471-2148-13-103 (PMC3680083; doi:10.1186/1471-2148-13-103)
Supplement: Additional file 3: Table S2 — Copy numbers of PHO1 genes in different classes in 30 land plant species. [file 1471-2148-13-103-S3.pptx]

## Slide 1
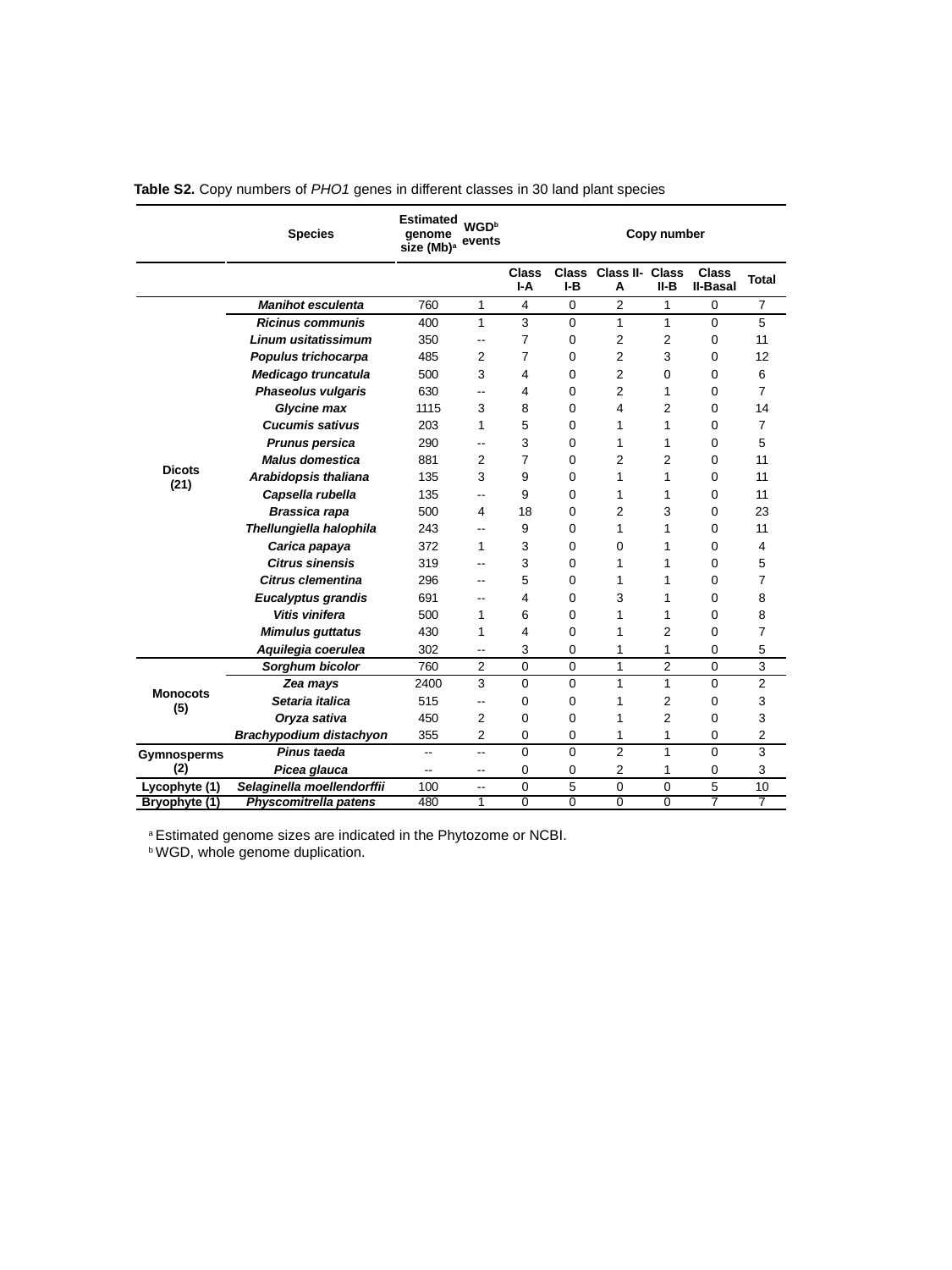

Table S2. Copy numbers of PHO1 genes in different classes in 30 land plant species
| | Species | Estimated genome size (Mb)a | WGDb events | Copy number | | | | | |
| --- | --- | --- | --- | --- | --- | --- | --- | --- | --- |
| | | | | Class I-A | Class I-B | Class II-A | Class II-B | Class II-Basal | Total |
| Dicots (21) | Manihot esculenta | 760 | 1 | 4 | 0 | 2 | 1 | 0 | 7 |
| | Ricinus communis | 400 | 1 | 3 | 0 | 1 | 1 | 0 | 5 |
| | Linum usitatissimum | 350 | -- | 7 | 0 | 2 | 2 | 0 | 11 |
| | Populus trichocarpa | 485 | 2 | 7 | 0 | 2 | 3 | 0 | 12 |
| | Medicago truncatula | 500 | 3 | 4 | 0 | 2 | 0 | 0 | 6 |
| | Phaseolus vulgaris | 630 | -- | 4 | 0 | 2 | 1 | 0 | 7 |
| | Glycine max | 1115 | 3 | 8 | 0 | 4 | 2 | 0 | 14 |
| | Cucumis sativus | 203 | 1 | 5 | 0 | 1 | 1 | 0 | 7 |
| | Prunus persica | 290 | -- | 3 | 0 | 1 | 1 | 0 | 5 |
| | Malus domestica | 881 | 2 | 7 | 0 | 2 | 2 | 0 | 11 |
| | Arabidopsis thaliana | 135 | 3 | 9 | 0 | 1 | 1 | 0 | 11 |
| | Capsella rubella | 135 | -- | 9 | 0 | 1 | 1 | 0 | 11 |
| | Brassica rapa | 500 | 4 | 18 | 0 | 2 | 3 | 0 | 23 |
| | Thellungiella halophila | 243 | -- | 9 | 0 | 1 | 1 | 0 | 11 |
| | Carica papaya | 372 | 1 | 3 | 0 | 0 | 1 | 0 | 4 |
| | Citrus sinensis | 319 | -- | 3 | 0 | 1 | 1 | 0 | 5 |
| | Citrus clementina | 296 | -- | 5 | 0 | 1 | 1 | 0 | 7 |
| | Eucalyptus grandis | 691 | -- | 4 | 0 | 3 | 1 | 0 | 8 |
| | Vitis vinifera | 500 | 1 | 6 | 0 | 1 | 1 | 0 | 8 |
| | Mimulus guttatus | 430 | 1 | 4 | 0 | 1 | 2 | 0 | 7 |
| | Aquilegia coerulea | 302 | -- | 3 | 0 | 1 | 1 | 0 | 5 |
| Monocots (5) | Sorghum bicolor | 760 | 2 | 0 | 0 | 1 | 2 | 0 | 3 |
| | Zea mays | 2400 | 3 | 0 | 0 | 1 | 1 | 0 | 2 |
| | Setaria italica | 515 | -- | 0 | 0 | 1 | 2 | 0 | 3 |
| | Oryza sativa | 450 | 2 | 0 | 0 | 1 | 2 | 0 | 3 |
| | Brachypodium distachyon | 355 | 2 | 0 | 0 | 1 | 1 | 0 | 2 |
| Gymnosperms (2) | Pinus taeda | -- | -- | 0 | 0 | 2 | 1 | 0 | 3 |
| | Picea glauca | -- | -- | 0 | 0 | 2 | 1 | 0 | 3 |
| Lycophyte (1) | Selaginella moellendorffii | 100 | -- | 0 | 5 | 0 | 0 | 5 | 10 |
| Bryophyte (1) | Physcomitrella patens | 480 | 1 | 0 | 0 | 0 | 0 | 7 | 7 |
a Estimated genome sizes are indicated in the Phytozome or NCBI.
b WGD, whole genome duplication.
