# Supplementary material for: Phylogeny, structural evolution and functional diversification of the plant PHOSPHATE1 gene family: a focus on Glycine max
Source: BMC Evol Biol. 2013 May 24;13:103. doi: 10.1186/1471-2148-13-103 (PMC3680083; doi:10.1186/1471-2148-13-103)
Supplement: Additional file 4: Table S3 — The presence of tandem duplications in Class IA. [file 1471-2148-13-103-S4.pptx]

## Slide 1
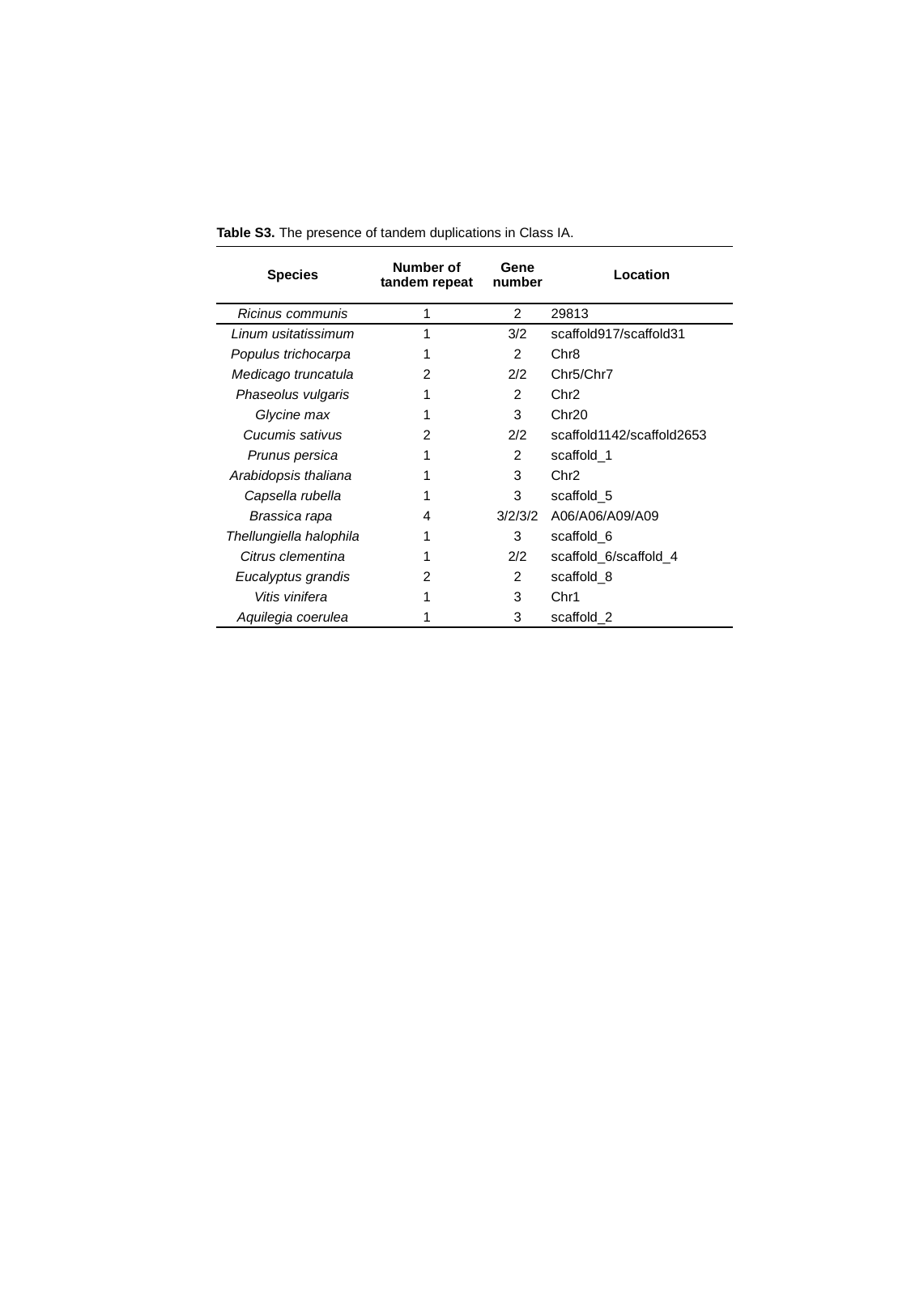

Table S3. The presence of tandem duplications in Class IA.
| Species | Number of tandem repeat | Gene number | Location |
| --- | --- | --- | --- |
| Ricinus communis | 1 | 2 | 29813 |
| Linum usitatissimum | 1 | 3/2 | scaffold917/scaffold31 |
| Populus trichocarpa | 1 | 2 | Chr8 |
| Medicago truncatula | 2 | 2/2 | Chr5/Chr7 |
| Phaseolus vulgaris | 1 | 2 | Chr2 |
| Glycine max | 1 | 3 | Chr20 |
| Cucumis sativus | 2 | 2/2 | scaffold1142/scaffold2653 |
| Prunus persica | 1 | 2 | scaffold\_1 |
| Arabidopsis thaliana | 1 | 3 | Chr2 |
| Capsella rubella | 1 | 3 | scaffold\_5 |
| Brassica rapa | 4 | 3/2/3/2 | A06/A06/A09/A09 |
| Thellungiella halophila | 1 | 3 | scaffold\_6 |
| Citrus clementina | 1 | 2/2 | scaffold\_6/scaffold\_4 |
| Eucalyptus grandis | 2 | 2 | scaffold\_8 |
| Vitis vinifera | 1 | 3 | Chr1 |
| Aquilegia coerulea | 1 | 3 | scaffold\_2 |
