# Supplementary material for: Phylogeny, structural evolution and functional diversification of the plant PHOSPHATE1 gene family: a focus on Glycine max
Source: BMC Evol Biol. 2013 May 24;13:103. doi: 10.1186/1471-2148-13-103 (PMC3680083; doi:10.1186/1471-2148-13-103)
Supplement: Additional file 5: Table S4 — Percent of members with different numbers of introns in each class. [file 1471-2148-13-103-S5.pptx]

## Slide 1
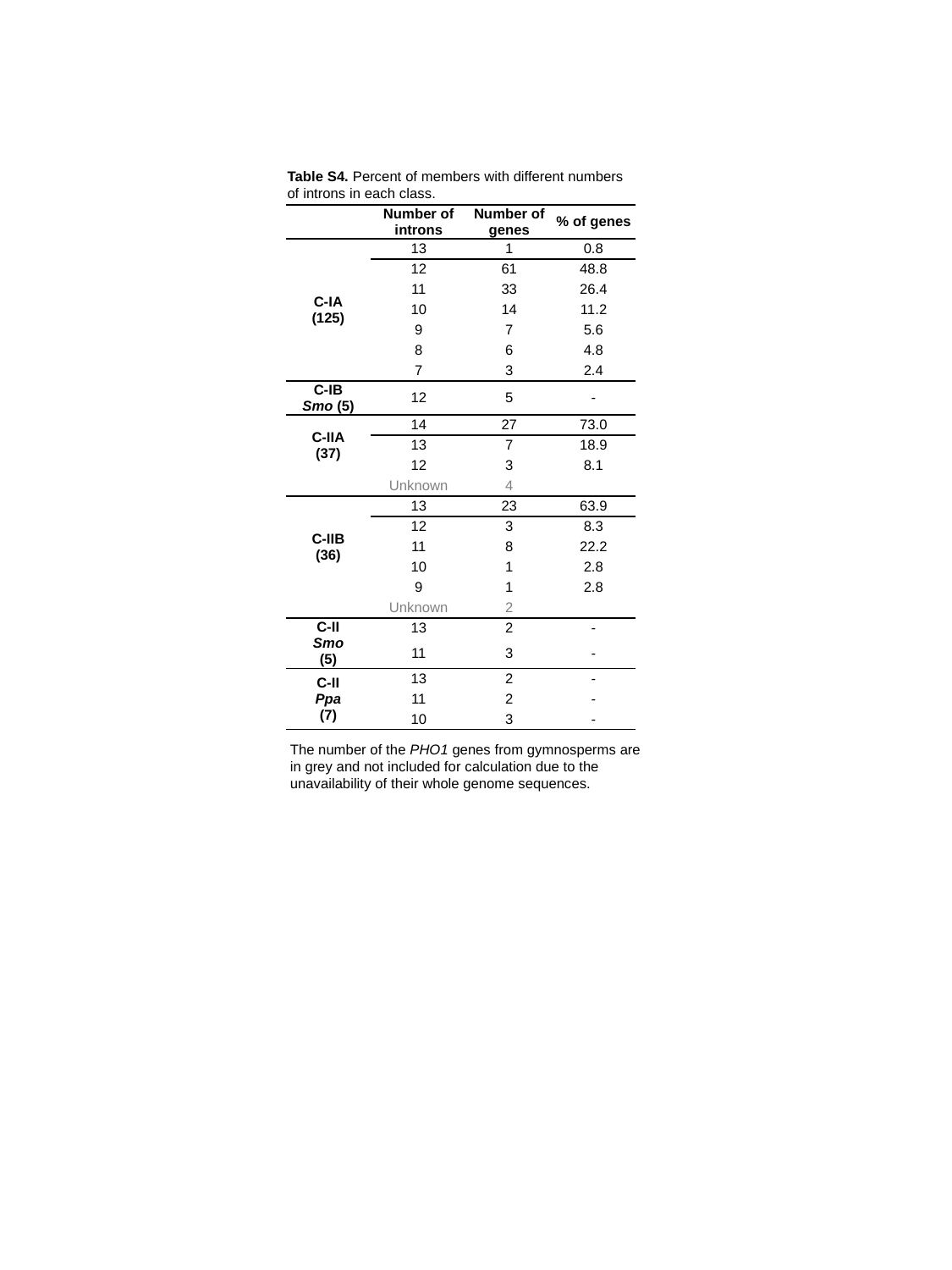

Table S4. Percent of members with different numbers of introns in each class.
| | Number of introns | Number of genes | % of genes |
| --- | --- | --- | --- |
| C-IA (125) | 13 | 1 | 0.8 |
| | 12 | 61 | 48.8 |
| | 11 | 33 | 26.4 |
| | 10 | 14 | 11.2 |
| | 9 | 7 | 5.6 |
| | 8 | 6 | 4.8 |
| | 7 | 3 | 2.4 |
| C-IB Smo (5) | 12 | 5 | - |
| C-IIA (37) | 14 | 27 | 73.0 |
| | 13 | 7 | 18.9 |
| | 12 | 3 | 8.1 |
| | Unknown | 4 | |
| C-IIB (36) | 13 | 23 | 63.9 |
| | 12 | 3 | 8.3 |
| | 11 | 8 | 22.2 |
| | 10 | 1 | 2.8 |
| | 9 | 1 | 2.8 |
| | Unknown | 2 | |
| C-II Smo (5) | 13 | 2 | - |
| | 11 | 3 | - |
| C-II Ppa (7) | 13 | 2 | - |
| | 11 | 2 | - |
| | 10 | 3 | - |
The number of the PHO1 genes from gymnosperms are in grey and not included for calculation due to the unavailability of their whole genome sequences.
