# Supplementary material for: Phylogeny, structural evolution and functional diversification of the plant PHOSPHATE1 gene family: a focus on Glycine max
Source: BMC Evol Biol. 2013 May 24;13:103. doi: 10.1186/1471-2148-13-103 (PMC3680083; doi:10.1186/1471-2148-13-103)
Supplement: Additional file 6: Figure S2 — Evolution of intron gain and loss events of the plant PHO1 gene family. ML tree of the plant PHO1 genes is identical to Figure1. Ancestral state reconstruction of PHO1 gene structure was conducted by Mesquite. Color scale depicts 19 different intron states of gene structures. Pie charts linked with the internal branches represent probability of ancestral states. Solid (one color) node indicates that the particular state is significantly better than all other possible states. Gene structures are based on the predicted sequences from the Phytozome database. The intron numbers corresponding to the assumed ancestral PHO1 genes are shown at the top. Numbers in boxes represent intron phases 0, 1 or 2, respectively. Black boxes indicate conserved introns, white boxes indicate lost introns, and gray boxes indicate inserted introns. [file 1471-2148-13-103-S6.pptx]

## Slide 1
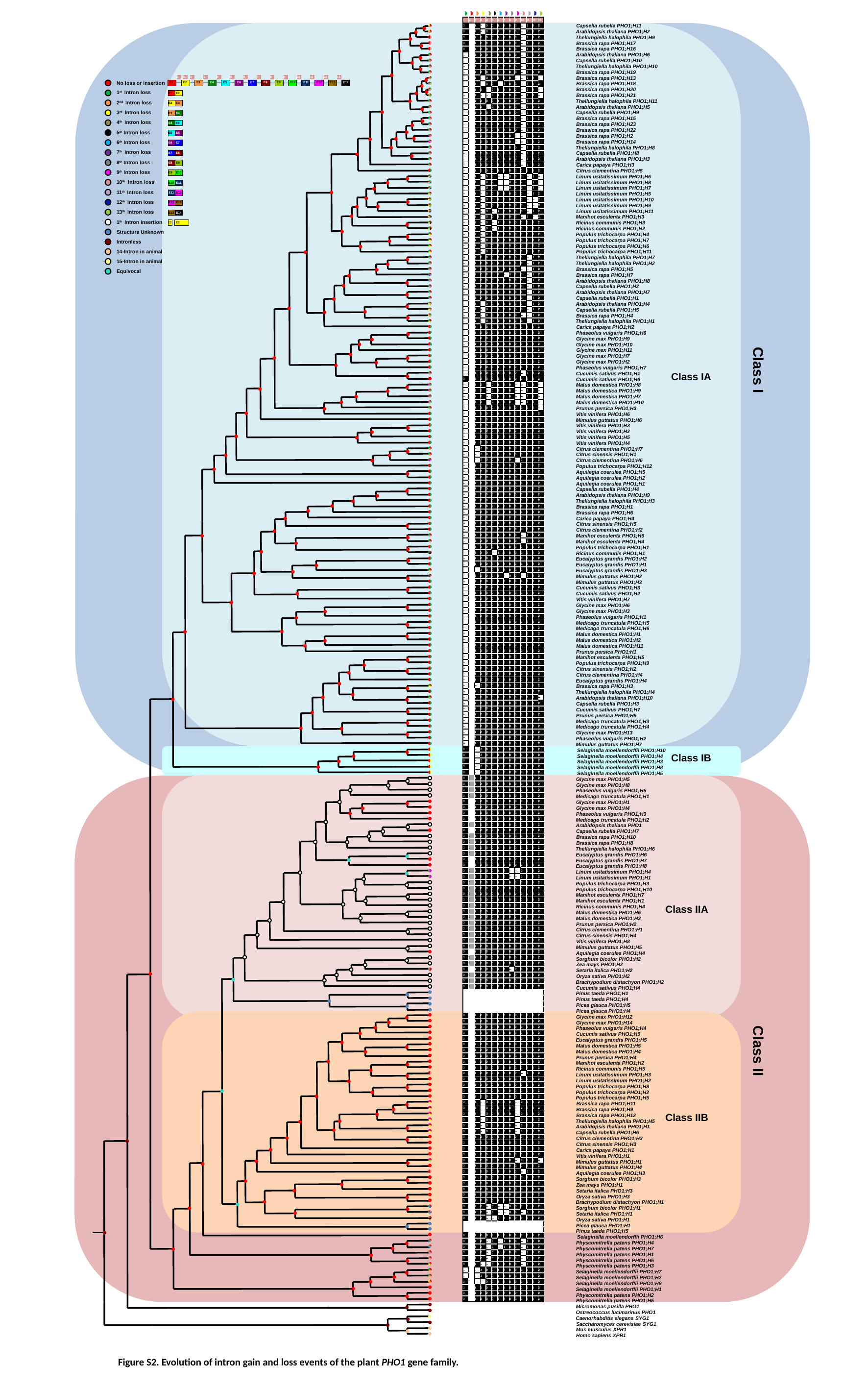

Capsella rubella PHO1;H11
Arabidopsis thaliana PHO1;H2
Thellungiella halophila PHO1;H9
Brassica rapa PHO1;H17
Brassica rapa PHO1;H16
Arabidopsis thaliana PHO1;H6
Capsella rubella PHO1;H10
Thellungiella halophila PHO1;H10
Brassica rapa PHO1;H19
Brassica rapa PHO1;H13
No loss or insertion
1st Intron loss
2nd Intron loss
3rd Intron loss
4th Intron loss
5th Intron loss
6th Intron loss
7th Intron loss
8th Intron loss
9th Intron loss
10th Intron loss
11th Intron loss
12th Intron loss
13th Intron loss
1th Intron insertion
Structure Unknown
14-Intron in animal
15-Intron in animal
Equivocal
Brassica rapa PHO1;H18
E1
E2
E3
E4
E5
E6
E7
E8
E9
E10
E11
E12
E13
E14
Brassica rapa PHO1;H20
E1
E2
Brassica rapa PHO1;H21
Thellungiella halophila PHO1;H11
E2
E3
Arabidopsis thaliana PHO1;H5
Capsella rubella PHO1;H9
E3
E4
Brassica rapa PHO1;H15
E4
E5
Brassica rapa PHO1;H23
Brassica rapa PHO1;H22
E5
E6
Brassica rapa PHO1;H2
Brassica rapa PHO1;H14
E6
E7
Thellungiella halophila PHO1;H8
Capsella rubella PHO1;H8
E7
E8
Arabidopsis thaliana PHO1;H3
E8
E9
Carica papaya PHO1;H3
Citrus clementina PHO1;H5
E9
E10
Linum usitatissimum PHO1;H6
Linum usitatissimum PHO1;H8
E10
E11
Linum usitatissimum PHO1;H7
Linum usitatissimum PHO1;H5
E11
E12
Linum usitatissimum PHO1;H10
E12
E13
Linum usitatissimum PHO1;H9
Linum usitatissimum PHO1;H11
E13
E14
Manihot esculenta PHO1;H3
Ricinus communis PHO1;H3
E2
E2
Ricinus communis PHO1;H2
Populus trichocarpa PHO1;H4
Populus trichocarpa PHO1;H7
Intronless
Populus trichocarpa PHO1;H6
Populus trichocarpa PHO1;H11
Thellungiella halophila PHO1;H7
Thellungiella halophila PHO1;H2
Brassica rapa PHO1;H5
Brassica rapa PHO1;H7
Arabidopsis thaliana PHO1;H8
Capsella rubella PHO1;H2
Arabidopsis thaliana PHO1;H7
Capsella rubella PHO1;H1
Arabidopsis thaliana PHO1;H4
Capsella rubella PHO1;H5
Brassica rapa PHO1;H4
Thellungiella halophila PHO1;H1
Carica papaya PHO1;H2
Phaseolus vulgaris PHO1;H6
Glycine max PHO1;H9
Glycine max PHO1;H10
Glycine max PHO1;H11
Glycine max PHO1;H7
Glycine max PHO1;H2
Phaseolus vulgaris PHO1;H7
Cucumis sativus PHO1;H1
Cucumis sativus PHO1;H6
Malus domestica PHO1;H8
Malus domestica PHO1;H9
Malus domestica PHO1;H7
Malus domestica PHO1;H10
Prunus persica PHO1;H3
Vitis vinifera PHO1;H6
Mimulus guttatus PHO1;H6
Vitis vinifera PHO1;H3
Vitis vinifera PHO1;H2
Vitis vinifera PHO1;H5
Vitis vinifera PHO1;H4
Citrus clementina PHO1;H7
Citrus sinensis PHO1;H1
Citrus clementina PHO1;H6
Populus trichocarpa PHO1;H12
Aquilegia coerulea PHO1;H5
Aquilegia coerulea PHO1;H2
Aquilegia coerulea PHO1;H1
Capsella rubella PHO1;H4
Arabidopsis thaliana PHO1;H9
Thellungiella halophila PHO1;H3
Brassica rapa PHO1;H1
Brassica rapa PHO1;H6
Carica papaya PHO1;H4
Citrus sinensis PHO1;H5
Citrus clementina PHO1;H2
Manihot esculenta PHO1;H6
Manihot esculenta PHO1;H4
Populus trichocarpa PHO1;H1
Ricinus communis PHO1;H1
Eucalyptus grandis PHO1;H2
Eucalyptus grandis PHO1;H1
Eucalyptus grandis PHO1;H3
Mimulus guttatus PHO1;H2
Mimulus guttatus PHO1;H3
Cucumis sativus PHO1;H3
Cucumis sativus PHO1;H2
Vitis vinifera PHO1;H7
Glycine max PHO1;H6
Glycine max PHO1;H3
Phaseolus vulgaris PHO1;H1
Medicago truncatula PHO1;H5
Medicago truncatula PHO1;H6
Malus domestica PHO1;H1
Malus domestica PHO1;H2
Malus domestica PHO1;H11
Prunus persica PHO1;H1
Manihot esculenta PHO1;H5
Populus trichocarpa PHO1;H9
Citrus sinensis PHO1;H2
Citrus clementina PHO1;H4
Eucalyptus grandis PHO1;H4
Brassica rapa PHO1;H3
Thellungiella halophila PHO1;H4
Arabidopsis thaliana PHO1;H10
Capsella rubella PHO1;H3
Cucumis sativus PHO1;H7
Prunus persica PHO1;H5
Medicago truncatula PHO1;H3
Medicago truncatula PHO1;H4
Glycine max PHO1;H13
Phaseolus vulgaris PHO1;H2
Mimulus guttatus PHO1;H7
Selaginella moellendorffii PHO1;H10
Selaginella moellendorffii PHO1;H4
Selaginella moellendorffii PHO1;H3
Selaginella moellendorffii PHO1;H8
Selaginella moellendorffii PHO1;H5
Glycine max PHO1;H5
Glycine max PHO1;H8
Phaseolus vulgaris PHO1;H5
Medicago truncatula PHO1;H1
Glycine max PHO1;H1
Glycine max PHO1;H4
Phaseolus vulgaris PHO1;H3
Medicago truncatula PHO1;H2
Arabidopsis thaliana PHO1
Capsella rubella PHO1;H7
Brassica rapa PHO1;H10
Brassica rapa PHO1;H8
Thellungiella halophila PHO1;H6
Eucalyptus grandis PHO1;H6
Eucalyptus grandis PHO1;H7
Eucalyptus grandis PHO1;H8
Linum usitatissimum PHO1;H4
Linum usitatissimum PHO1;H1
Populus trichocarpa PHO1;H3
Populus trichocarpa PHO1;H10
Manihot esculenta PHO1;H7
Manihot esculenta PHO1;H1
Ricinus communis PHO1;H4
Malus domestica PHO1;H6
Malus domestica PHO1;H3
Prunus persica PHO1;H2
Citrus clementina PHO1;H1
Citrus sinensis PHO1;H4
Vitis vinifera PHO1;H8
Mimulus guttatus PHO1;H5
Aquilegia coerulea PHO1;H4
Sorghum bicolor PHO1;H2
Zea mays PHO1;H2
Setaria italica PHO1;H2
Oryza sativa PHO1;H2
Brachypodium distachyon PHO1;H2
Cucumis sativus PHO1;H4
Pinus taeda PHO1;H1
Pinus taeda PHO1;H4
Picea glauca PHO1;H5
Picea glauca PHO1;H4
Glycine max PHO1;H12
Glycine max PHO1;H14
Phaseolus vulgaris PHO1;H4
Cucumis sativus PHO1;H5
Eucalyptus grandis PHO1;H5
Malus domestica PHO1;H5
Malus domestica PHO1;H4
Prunus persica PHO1;H4
Manihot esculenta PHO1;H2
Ricinus communis PHO1;H5
Linum usitatissimum PHO1;H3
Linum usitatissimum PHO1;H2
Populus trichocarpa PHO1;H8
Populus trichocarpa PHO1;H2
Populus trichocarpa PHO1;H5
Brassica rapa PHO1;H11
Brassica rapa PHO1;H9
Brassica rapa PHO1;H12
Thellungiella halophila PHO1;H5
Arabidopsis thaliana PHO1;H1
Capsella rubella PHO1;H6
Citrus clementina PHO1;H3
Citrus sinensis PHO1;H3
Carica papaya PHO1;H1
Vitis vinifera PHO1;H1
Mimulus guttatus PHO1;H1
Mimulus guttatus PHO1;H4
Aquilegia coerulea PHO1;H3
Sorghum bicolor PHO1;H3
Zea mays PHO1;H1
Setaria italica PHO1;H3
Oryza sativa PHO1;H3
Brachypodium distachyon PHO1;H1
Sorghum bicolor PHO1;H1
Setaria italica PHO1;H1
Oryza sativa PHO1;H1
Picea glauca PHO1;H1
Pinus taeda PHO1;H5
Selaginella moellendorffii PHO1;H6
Physcomitrella patens PHO1;H4
Physcomitrella patens PHO1;H7
Physcomitrella patens PHO1;H1
Physcomitrella patens PHO1;H6
Physcomitrella patens PHO1;H3
Selaginella moellendorffii PHO1;H7
Selaginella moellendorffii PHO1;H2
Selaginella moellendorffii PHO1;H9
Selaginella moellendorffii PHO1;H1
Physcomitrella patens PHO1;H2
Physcomitrella patens PHO1;H5
Micromonas pusilla PHO1
Ostreococcus lucimarinus PHO1
Caenorhabditis elegans SYG1
Saccharomyces cerevisiae SYG1
Mus musculus XPR1
Homo sapiens XPR1
| 1 | 1i | 2 | 3 | 4 | 5 | 6 | 7 | 8 | 9 | 10 | 11 | 12 | 13 |
| --- | --- | --- | --- | --- | --- | --- | --- | --- | --- | --- | --- | --- | --- |
| 0 | | 1 | -- | 0 | 0 | 1 | 2 | 0 | 0 | -- | 0 | 0 | 2 |
| 0 | | 1 | -- | 0 | 0 | 1 | 2 | 0 | 0 | -- | 0 | 0 | 2 |
| 0 | | 1 | 2 | 0 | 0 | 1 | 2 | 0 | 0 | -- | 0 | 0 | 2 |
| 0 | | 1 | 2 | 0 | 0 | 1 | 2 | 0 | 0 | -- | 0 | 0 | 2 |
| 0 | | 1 | 2 | 0 | 0 | 1 | 2 | 0 | 0 | -- | 0 | 0 | 2 |
| -- | | 1 | 2 | 0 | 0 | 1 | 2 | 0 | 0 | -- | 0 | 0 | 2 |
| -- | | 1 | 2 | 0 | 0 | 1 | 2 | 0 | 0 | -- | 0 | 0 | 2 |
| -- | | 1 | 2 | 0 | 0 | 1 | 2 | 0 | 0 | -- | 0 | 0 | 2 |
| -- | | 1 | 2 | 0 | 0 | 1 | 2 | 0 | 0 | -- | 0 | 0 | 2 |
| -- | | 1 | -- | 0 | 0 | 1 | 2 | 0 | -- | -- | 0 | 0 | -- |
| -- | | 1 | -- | 0 | 0 | -- | 2 | 0 | 0 | -- | 0 | 0 | 2 |
| -- | | 1 | 2 | -- | 0 | 1 | 2 | 0 | 0 | -- | 0 | 0 | -- |
| -- | | 1 | -- | -- | 0 | 1 | 2 | 0 | 0 | -- | 0 | 0 | -- |
| -- | | 1 | 2 | 0 | 0 | 1 | 2 | 0 | 0 | -- | 0 | 0 | 2 |
| -- | | 1 | 2 | -- | 0 | 1 | 2 | 0 | 0 | -- | 0 | 0 | 2 |
| -- | | 1 | 2 | 0 | 0 | 1 | 2 | 0 | 0 | -- | 0 | 0 | 2 |
| -- | | 1 | 2 | 0 | 0 | 1 | 2 | 0 | 0 | -- | 0 | 0 | 2 |
| -- | | 1 | 2 | 0 | 0 | 1 | 2 | 0 | 0 | -- | 0 | 0 | 2 |
| -- | | 1 | 2 | 0 | 0 | 1 | 2 | 0 | 0 | -- | 0 | 0 | 2 |
| -- | | 1 | 2 | 0 | 0 | 1 | 2 | 0 | -- | -- | 0 | 0 | 2 |
| -- | | 1 | 2 | 0 | 0 | 1 | 2 | 0 | -- | -- | 0 | 0 | 2 |
| -- | | 1 | 2 | 0 | 0 | 1 | 2 | 0 | 0 | -- | 0 | 0 | 2 |
| -- | | 1 | 2 | 0 | 0 | 1 | 2 | 0 | 0 | -- | 0 | 0 | 2 |
| -- | | 1 | 2 | 0 | 0 | 1 | 2 | 0 | 0 | -- | 0 | 0 | 2 |
| -- | | 1 | 2 | 0 | 0 | 1 | 2 | 0 | 0 | -- | 0 | 0 | 2 |
| -- | | 1 | 2 | 0 | 0 | 1 | 2 | 0 | 0 | 0 | 0 | 0 | 2 |
| -- | | 1 | -- | 0 | 0 | -- | -- | 0 | 0 | 0 | -- | 0 | -- |
| -- | | 1 | -- | 0 | 0 | -- | -- | 0 | 0 | 0 | -- | 0 | -- |
| -- | | 1 | -- | 0 | 0 | -- | -- | 0 | 0 | 0 | -- | 0 | -- |
| -- | | 1 | -- | 0 | 0 | 1 | 2 | 0 | 0 | 0 | -- | 0 | 2 |
| -- | | 1 | -- | 0 | 0 | 1 | 2 | 0 | 0 | 0 | -- | -- | 2 |
| -- | | 1 | -- | 0 | 0 | 1 | 2 | 0 | 0 | 0 | -- | -- | 2 |
| -- | | 1 | -- | 0 | -- | 1 | 2 | 0 | 0 | 0 | -- | 0 | 2 |
| -- | | 1 | -- | 0 | 0 | 1 | 2 | 0 | 0 | -- | 0 | -- | 2 |
| -- | | 1 | -- | 0 | -- | 1 | 2 | 0 | 0 | 0 | 0 | 0 | 2 |
| -- | | 1 | -- | 0 | -- | 1 | 2 | 0 | 0 | 0 | 0 | 0 | 2 |
| -- | | 1 | -- | 0 | 0 | 1 | 2 | 0 | 0 | 0 | 0 | 0 | 2 |
| -- | | 1 | -- | 0 | 0 | 1 | 2 | 0 | 0 | 0 | 0 | 0 | 2 |
| -- | | 1 | -- | 0 | 0 | 1 | 2 | 0 | 0 | 0 | 0 | 0 | 2 |
| -- | | 1 | -- | 0 | 0 | 1 | 2 | 0 | 0 | 0 | 0 | 0 | 2 |
| -- | | 1 | 2 | 0 | 0 | 1 | 2 | 0 | 0 | 0 | -- | 0 | 2 |
| -- | | 1 | 2 | 0 | 0 | 1 | 2 | 0 | 0 | 0 | -- | 0 | 2 |
| -- | | 1 | 2 | 0 | 0 | 1 | 2 | 0 | 0 | -- | -- | 0 | 2 |
| -- | | 1 | 2 | 0 | 0 | 1 | -- | 0 | 0 | 0 | -- | 0 | 2 |
| -- | | 1 | 2 | 0 | 0 | 1 | 2 | 0 | 0 | 0 | -- | 0 | 2 |
| -- | | 1 | 2 | 0 | 0 | 1 | 2 | 0 | 0 | 0 | -- | 0 | 2 |
| -- | | 1 | 2 | 0 | 0 | 1 | 2 | 0 | 0 | 0 | -- | 0 | 2 |
| -- | | 1 | 2 | 0 | 0 | 1 | 2 | 0 | 0 | 0 | -- | 0 | 2 |
| -- | | 1 | -- | 0 | 0 | 1 | 2 | 0 | 0 | 0 | -- | 0 | 2 |
| -- | | 1 | -- | 0 | 0 | 1 | 2 | 0 | 0 | 0 | -- | 0 | 2 |
| -- | | 1 | -- | 0 | 0 | 1 | 2 | 0 | 0 | -- | -- | 0 | 2 |
| -- | | 1 | -- | 0 | 0 | 1 | 2 | 0 | 0 | 0 | -- | 0 | 2 |
| -- | | 1 | 2 | 0 | 0 | 1 | 2 | 0 | 0 | 0 | 0 | 0 | 2 |
| -- | | 1 | 2 | 0 | 0 | 1 | 2 | 0 | 0 | 0 | 0 | 0 | 2 |
| -- | | 1 | 2 | 0 | 0 | 1 | 2 | 0 | 0 | 0 | 0 | 0 | 2 |
| -- | | 1 | 2 | 0 | 0 | 1 | 2 | 0 | 0 | 0 | 0 | 0 | 2 |
| -- | | 1 | 2 | 0 | 0 | 1 | 2 | 0 | 0 | 0 | 0 | 0 | 2 |
| -- | | 1 | 2 | 0 | 0 | 1 | 2 | 0 | 0 | 0 | 0 | 0 | 2 |
| -- | | 1 | 2 | 0 | 0 | 1 | 2 | 0 | 0 | 0 | 0 | 0 | 2 |
| -- | | 1 | 2 | 0 | 0 | 1 | 2 | 0 | 0 | 0 | 0 | 0 | 2 |
| -- | | 1 | 2 | 0 | 0 | 1 | 2 | 0 | 0 | -- | 0 | 0 | 2 |
| 0 | | 1 | 2 | 0 | 0 | 1 | 2 | 0 | 0 | 0 | 0 | 0 | 2 |
| -- | | 1 | 2 | -- | 0 | 1 | 2 | 0 | -- | -- | 0 | 0 | -- |
| -- | | 1 | 2 | -- | 0 | 1 | 2 | 0 | -- | -- | 0 | 0 | -- |
| -- | | 1 | 2 | -- | 0 | 1 | 2 | 0 | -- | -- | 0 | 0 | -- |
| -- | | 1 | 2 | -- | 0 | 1 | 2 | 0 | -- | -- | 0 | 0 | -- |
| -- | | 1 | 2 | 0 | 0 | 1 | 2 | 0 | 0 | 0 | 0 | 0 | -- |
| -- | | 1 | 2 | 0 | 0 | 1 | 2 | 0 | 0 | 0 | 0 | 0 | 2 |
| -- | | 1 | 2 | 0 | 0 | 1 | 2 | 0 | 0 | 0 | 0 | 0 | 2 |
| -- | | 1 | 2 | 0 | 0 | 1 | 2 | 0 | 0 | 0 | 0 | 0 | 2 |
| -- | | 1 | 2 | 0 | 0 | 1 | 2 | 0 | 0 | 0 | 0 | 0 | 2 |
| -- | | 1 | 2 | 0 | 0 | 1 | 2 | 0 | 0 | 0 | 0 | 0 | 2 |
| -- | | 1 | 2 | 0 | 0 | 1 | 2 | 0 | 0 | 0 | 0 | 0 | 2 |
| -- | | -- | 2 | 0 | 0 | 1 | 2 | 0 | 0 | 0 | 0 | 0 | 2 |
| -- | | -- | 2 | 0 | 0 | 1 | 2 | 0 | 0 | 0 | 0 | 0 | 2 |
| -- | | -- | 2 | 0 | 0 | 1 | 2 | 0 | -- | 0 | 0 | 0 | 2 |
| -- | | 1 | 2 | 0 | 0 | 1 | 2 | 0 | 0 | 0 | 0 | 0 | 2 |
| -- | | 1 | 2 | 0 | 0 | 1 | 2 | 0 | 0 | 0 | 0 | 0 | 2 |
| -- | | 1 | 2 | 0 | 0 | 1 | 2 | 0 | 0 | 0 | 0 | 0 | 2 |
| -- | | 1 | 2 | 0 | 0 | 1 | 2 | 0 | 0 | 0 | 0 | 0 | 2 |
| -- | | 1 | 2 | 0 | 0 | 1 | 2 | 0 | 0 | 0 | 0 | 0 | 2 |
| -- | | 1 | 2 | 0 | 0 | 1 | 2 | 0 | 0 | 0 | 0 | 0 | 2 |
| -- | | 1 | 2 | 0 | 0 | 1 | 2 | 0 | 0 | 0 | 0 | 0 | 2 |
| -- | | 1 | 2 | 0 | 0 | 1 | 2 | 0 | 0 | 0 | 0 | 0 | 2 |
| -- | | 1 | 2 | 0 | 0 | 1 | 2 | 0 | 0 | 0 | 0 | 0 | 2 |
| -- | | 1 | 2 | 0 | 0 | 1 | 2 | 0 | 0 | 0 | 0 | 0 | 2 |
| -- | | 1 | 2 | 0 | 0 | 1 | 2 | 0 | 0 | 0 | 0 | 0 | 2 |
| -- | | 1 | 2 | 0 | 0 | 1 | 2 | 0 | 0 | 0 | 0 | 0 | 2 |
| -- | | 1 | 2 | 0 | 0 | 1 | 2 | 0 | 0 | -- | 0 | 0 | 2 |
| -- | | 1 | 2 | 0 | 0 | 1 | 2 | 0 | 0 | -- | 0 | 0 | 2 |
| -- | | 1 | 2 | 0 | 0 | 1 | 2 | 0 | 0 | 0 | 0 | 0 | 2 |
| -- | | 1 | 2 | 0 | -- | 1 | 2 | 0 | 0 | 0 | 0 | 0 | 2 |
| -- | | 1 | 2 | 0 | 0 | 1 | 2 | 0 | 0 | 0 | 0 | 0 | 2 |
| -- | | 1 | 2 | 0 | 0 | 1 | 2 | 0 | 0 | 0 | 0 | 0 | 2 |
| -- | | -- | 2 | 0 | 0 | 1 | 2 | 0 | 0 | 0 | 0 | 0 | 2 |
| -- | | 1 | 2 | 0 | 0 | 1 | -- | 0 | 0 | -- | 0 | 0 | 2 |
| -- | | 1 | 2 | 0 | 0 | 1 | 2 | 0 | 0 | 0 | 0 | 0 | 2 |
| -- | | 1 | 2 | 0 | 0 | 1 | 2 | 0 | 0 | 0 | 0 | 0 | 2 |
| -- | | 1 | 2 | 0 | 0 | 1 | 2 | 0 | 0 | 0 | 0 | 0 | 2 |
| -- | | 1 | 2 | 0 | 0 | 1 | 2 | 0 | 0 | 0 | 0 | 0 | 2 |
| -- | | 1 | 2 | 0 | 0 | 1 | 2 | 0 | 0 | 0 | 0 | 0 | 2 |
| -- | | 1 | 2 | 0 | 0 | 1 | 2 | 0 | 0 | 0 | 0 | 0 | 2 |
| -- | | 1 | 2 | 0 | 0 | 1 | 2 | 0 | 0 | 0 | 0 | 0 | 2 |
| -- | | 1 | 2 | 0 | 0 | 1 | 2 | 0 | 0 | 0 | 0 | 0 | 2 |
| -- | | 1 | 2 | 0 | 0 | 1 | 2 | 0 | 0 | 0 | 0 | 0 | 2 |
| -- | | 1 | 2 | 0 | 0 | 1 | 2 | 0 | 0 | 0 | 0 | 0 | 2 |
| -- | | 1 | 2 | 0 | 0 | 1 | 2 | 0 | 0 | 0 | 0 | 0 | 2 |
| -- | | 1 | 2 | 0 | 0 | 1 | 2 | 0 | 0 | 0 | 0 | 0 | 2 |
| -- | | 1 | 2 | 0 | 0 | 1 | 2 | 0 | 0 | 0 | 0 | 0 | 2 |
| -- | | 1 | 2 | 0 | 0 | 1 | 2 | 0 | 0 | 0 | 0 | 0 | 2 |
| -- | | 1 | 2 | 0 | 0 | 1 | 2 | 0 | 0 | 0 | 0 | 0 | 2 |
| -- | | 1 | 2 | 0 | 0 | 1 | 2 | 0 | 0 | 0 | 0 | 0 | 2 |
| -- | | 1 | 2 | 0 | 0 | 1 | 2 | 0 | 0 | 0 | 0 | 0 | 2 |
| -- | | 1 | 2 | 0 | 0 | 1 | 2 | 0 | 0 | 0 | 0 | 0 | 2 |
| -- | | -- | 2 | 0 | 0 | 1 | 2 | 0 | 0 | 0 | 0 | 0 | 2 |
| -- | | 1 | 2 | 0 | 0 | 1 | 2 | 0 | 0 | 0 | 0 | 0 | 2 |
| -- | | 1 | 2 | 0 | 0 | 1 | 2 | 0 | 0 | 0 | 0 | 0 | -- |
| -- | | 1 | 2 | 0 | 0 | 1 | 2 | 0 | 0 | 0 | 0 | 0 | 2 |
| -- | | 1 | 2 | 0 | 0 | 1 | 2 | 0 | 0 | 0 | 0 | 0 | 2 |
| -- | | 1 | 2 | 0 | 0 | 1 | 2 | 0 | 0 | 0 | 0 | 0 | 2 |
| -- | | 1 | 2 | 0 | 0 | 1 | 2 | 0 | 0 | 0 | 0 | 0 | 2 |
| -- | | 1 | 2 | 0 | 0 | 1 | 2 | 0 | 0 | 0 | 0 | 0 | 2 |
| -- | | 1 | 2 | 0 | 0 | 1 | 2 | 0 | 0 | 0 | 0 | 0 | 2 |
| -- | | 1 | 2 | 0 | 0 | 1 | 2 | 0 | 0 | 0 | 0 | 0 | 2 |
| -- | | 1 | 2 | 0 | 0 | 1 | 2 | 0 | 0 | 0 | 0 | 0 | 2 |
| 0 | | -- | 2 | 0 | 0 | 1 | 2 | 0 | 0 | 0 | 0 | 0 | 2 |
| 0 | | -- | 2 | 0 | 0 | 1 | 2 | 0 | 0 | 0 | 0 | 0 | 2 |
| 0 | | -- | 2 | 0 | 0 | 1 | 2 | 0 | 0 | 0 | 0 | 0 | 2 |
| 0 | | -- | 2 | 0 | 0 | 1 | 2 | 0 | 0 | 0 | 0 | 0 | 2 |
| 0 | | -- | 2 | 0 | 0 | 1 | 2 | 0 | 0 | 0 | 0 | 0 | 2 |
| 0 | 0 | 1 | 2 | 0 | 0 | 1 | 2 | 0 | 0 | 0 | 0 | 0 | 2 |
| 0 | 0 | 1 | 2 | 0 | 0 | 1 | 2 | 0 | 0 | 0 | 0 | 0 | 2 |
| 0 | 0 | 1 | 2 | 0 | 0 | 1 | 2 | 0 | 0 | 0 | 0 | 0 | 2 |
| 0 | 0 | 1 | 2 | 0 | 0 | 1 | 2 | 0 | 0 | 0 | 0 | 0 | 2 |
| 0 | | 1 | 2 | 0 | 0 | 1 | 2 | 0 | 0 | 0 | 0 | 0 | 2 |
| 0 | | 1 | 2 | 0 | 0 | 1 | 2 | 0 | 0 | 0 | 0 | 0 | 2 |
| 0 | | 1 | 2 | 0 | 0 | 1 | 2 | 0 | 0 | 0 | 0 | 0 | 2 |
| 0 | | 1 | 2 | 0 | 0 | 1 | 2 | 0 | 0 | 0 | 0 | 0 | 2 |
| 0 | 0 | 1 | 2 | 0 | 0 | 1 | 2 | 0 | 0 | 0 | 0 | 0 | 2 |
| 0 | | 1 | 2 | 0 | 0 | 1 | 2 | 0 | 0 | 0 | 0 | 0 | 2 |
| 0 | 0 | 1 | 2 | 0 | 0 | 1 | 2 | 0 | 0 | 0 | 0 | 0 | 2 |
| 0 | 0 | 1 | 2 | 0 | 0 | 1 | 2 | 0 | 0 | 0 | 0 | 0 | 2 |
| 0 | 0 | 1 | 2 | 0 | 0 | 1 | 2 | 0 | 0 | 0 | 0 | 0 | 2 |
| 0 | 0 | 1 | 2 | 0 | 0 | 1 | 2 | 0 | 0 | 0 | 0 | 0 | 2 |
| 0 | | 1 | 2 | 0 | 0 | 1 | 2 | 0 | 0 | 0 | 0 | 0 | 2 |
| 0 | | 1 | 2 | 0 | 0 | 1 | 2 | 0 | 0 | 0 | 0 | 0 | 2 |
| 0 | 0 | 1 | 2 | 0 | 0 | 1 | 2 | -- | -- | 0 | 0 | 0 | 2 |
| 0 | 0 | 1 | 2 | 0 | 0 | 1 | 2 | -- | -- | 0 | 0 | 0 | 2 |
| 0 | 0 | 1 | 2 | 0 | 0 | 1 | 2 | 0 | 0 | 0 | 0 | 0 | 2 |
| 0 | 0 | 1 | 2 | 0 | 0 | 1 | 2 | 0 | 0 | 0 | 0 | 0 | 2 |
| 0 | 0 | 1 | 2 | 0 | 0 | 1 | 2 | 0 | 0 | 0 | 0 | 0 | 2 |
| 0 | 0 | 1 | 2 | 0 | 0 | 1 | 2 | 0 | 0 | 0 | 0 | 0 | 2 |
| 0 | 0 | 1 | 2 | 0 | 0 | 1 | 2 | 0 | 0 | 0 | 0 | 0 | 2 |
| 0 | 0 | 1 | 2 | 0 | 0 | 1 | 2 | 0 | 0 | 0 | 0 | 0 | 2 |
| 0 | 0 | 1 | 2 | 0 | 0 | 1 | 2 | 0 | 0 | 0 | 0 | 0 | 2 |
| 0 | 0 | 1 | 2 | 0 | 0 | 1 | 2 | 0 | 0 | 0 | 0 | 0 | 2 |
| 0 | 0 | 1 | 2 | 0 | 0 | 1 | 2 | 0 | 0 | 0 | 0 | 0 | 2 |
| 0 | 0 | 1 | 2 | 0 | 0 | 1 | 2 | 0 | 0 | 0 | 0 | 0 | 2 |
| 0 | 0 | 1 | 2 | 0 | 0 | 1 | 2 | 0 | 0 | 0 | 0 | 0 | 2 |
| 0 | 0 | 1 | 2 | 0 | 0 | 1 | 2 | 0 | 0 | 0 | 0 | 0 | 2 |
| 0 | | 1 | 2 | 0 | 0 | 1 | 2 | 0 | 0 | 0 | 0 | 0 | 2 |
| 0 | 0 | 1 | 2 | 0 | 0 | 1 | 2 | 0 | 0 | 0 | 0 | 0 | 2 |
| 0 | 0 | 1 | 2 | 0 | 0 | 1 | 2 | 0 | 0 | 0 | 0 | 0 | 2 |
| 0 | | 1 | 2 | 0 | 0 | 1 | 2 | -- | 0 | 0 | 0 | 0 | 2 |
| 0 | 0 | 1 | 2 | 0 | 0 | 1 | 2 | 0 | 0 | 0 | 0 | 0 | 2 |
| 0 | 0 | 1 | 2 | 0 | 0 | 1 | 2 | 0 | 0 | 0 | 0 | 0 | 2 |
| 0 | 0 | 1 | 2 | 0 | 0 | 1 | 2 | 0 | 0 | 0 | 0 | 0 | 2 |
| | | | | | | | | | | | | | |
| | | | | | | | | | | | | | |
| | | | | | | | | | | | | | |
| | | | | | | | | | | | | | |
| 0 | | 1 | 2 | 0 | 0 | 1 | 2 | 0 | 0 | 0 | 0 | 0 | 2 |
| 0 | | 1 | 2 | 0 | 0 | 1 | 2 | 0 | 0 | 0 | 0 | 0 | 2 |
| 0 | | 1 | 2 | 0 | 0 | 1 | 2 | 0 | 0 | 0 | 0 | 0 | 2 |
| 0 | | 1 | 2 | 0 | 0 | 1 | 2 | 0 | 0 | 0 | 0 | 0 | 2 |
| 0 | | 1 | 2 | 0 | 0 | 1 | 2 | 0 | 0 | 0 | 0 | 0 | 2 |
| 0 | | 1 | 2 | 0 | 0 | 1 | 2 | 0 | 0 | 0 | 0 | 0 | 2 |
| 0 | | 1 | 2 | 0 | 0 | 1 | 2 | 0 | 0 | 0 | 0 | 0 | 2 |
| 0 | | 1 | 2 | 0 | 0 | 1 | 2 | 0 | 0 | 0 | 0 | 0 | 2 |
| 0 | | 1 | 2 | 0 | 0 | 1 | 2 | 0 | 0 | 0 | 0 | 0 | 2 |
| 0 | | 1 | 2 | 0 | 0 | 1 | 2 | 0 | 0 | 0 | 0 | 0 | 2 |
| 0 | | 1 | 2 | 0 | 0 | 1 | 2 | 0 | 0 | -- | 0 | 0 | 2 |
| 0 | | 1 | 2 | 0 | 0 | 1 | 2 | 0 | 0 | 0 | 0 | 0 | 2 |
| 0 | | 1 | 2 | 0 | 0 | 1 | 2 | 0 | 0 | 0 | 0 | 0 | 2 |
| 0 | | 1 | 2 | 0 | 0 | 1 | 2 | 0 | 0 | 0 | 0 | 0 | 2 |
| 0 | | 1 | 2 | 0 | 0 | 1 | 2 | 0 | 0 | 0 | 0 | 0 | 2 |
| 0 | | 1 | -- | 0 | 0 | 1 | 2 | 0 | -- | 0 | 0 | 0 | 2 |
| 0 | | 1 | -- | 0 | 0 | 1 | 2 | 0 | -- | 0 | 0 | 0 | 2 |
| 0 | | 1 | -- | 0 | 0 | 1 | 2 | 0 | -- | 0 | 0 | 0 | 2 |
| 0 | | 1 | -- | 0 | 0 | 1 | 2 | 0 | -- | 0 | 0 | 0 | 2 |
| 0 | | 1 | -- | 0 | 0 | 1 | 2 | 0 | -- | 0 | 0 | 0 | 2 |
| 0 | | 1 | -- | 0 | 0 | 1 | 2 | 0 | -- | 0 | 0 | 0 | 2 |
| 0 | | 1 | 2 | 0 | 0 | 1 | 2 | 0 | 0 | 0 | 0 | 0 | 2 |
| 0 | | 1 | 2 | 0 | 0 | 1 | 2 | 0 | 0 | 0 | 0 | 0 | 2 |
| 0 | | 1 | 2 | 0 | 0 | 1 | 2 | 0 | 0 | 0 | 0 | 0 | 2 |
| 0 | | 1 | 2 | 0 | 0 | 1 | 2 | 0 | 0 | 0 | 0 | 0 | 2 |
| 0 | | 1 | 2 | 0 | 0 | 1 | 2 | 0 | -- | 0 | 0 | 0 | -- |
| 0 | | 1 | 2 | 0 | 0 | 1 | 2 | 0 | 0 | 0 | 0 | 0 | 2 |
| 0 | | 1 | 2 | 0 | 0 | 1 | 2 | 0 | 0 | -- | 0 | 0 | 2 |
| 0 | | 1 | 2 | 0 | 0 | 1 | 2 | 0 | 0 | 0 | 0 | 0 | 2 |
| 0 | | 1 | 2 | 0 | 0 | 1 | 2 | 0 | 0 | 0 | 0 | 0 | 2 |
| 0 | | 1 | 2 | 0 | 0 | 1 | 2 | 0 | 0 | 0 | 0 | 0 | 2 |
| 0 | | 1 | 2 | 0 | 0 | 1 | 2 | 0 | 0 | 0 | 0 | 0 | 2 |
| 0 | | 1 | 2 | 0 | 0 | 1 | 2 | 0 | 0 | 0 | 0 | 0 | 2 |
| 0 | | 1 | 2 | -- | 0 | -- | -- | 0 | 0 | 0 | 0 | 0 | 2 |
| 0 | | 1 | 2 | -- | 0 | -- | -- | 0 | 0 | -- | 0 | 0 | 2 |
| 0 | | 1 | 2 | -- | -- | 0 | 2 | 0 | 0 | 0 | 0 | 0 | 2 |
| | | | | | | | | | | | | | |
| | | | | | | | | | | | | | |
| 0 | | 1 | 2 | 0 | 0 | 1 | 2 | 0 | 0 | 0 | 0 | 0 | 2 |
| 0 | | 1 | 2 | -- | 0 | -- | 1 | 0 | 0 | -- | 0 | 0 | 2 |
| 0 | | 1 | 2 | -- | 0 | -- | 1 | 0 | 0 | -- | 0 | 0 | 2 |
| 0 | | 1 | 2 | -- | 0 | 1 | 2 | 0 | 0 | -- | 0 | 0 | 2 |
| 0 | | 1 | 2 | -- | 0 | 1 | 2 | 0 | 0 | -- | 0 | 0 | 2 |
| 0 | | 1 | -- | -- | 0 | 1 | 2 | 0 | 0 | -- | 0 | 0 | 2 |
| -- | | -- | 2 | 0 | 0 | 1 | 2 | 0 | 0 | 0 | 0 | 0 | 2 |
| -- | | -- | 2 | 0 | 0 | 1 | 2 | 0 | 0 | 0 | 0 | 0 | 2 |
| 0 | | -- | -- | 0 | 0 | 1 | 2 | 0 | 0 | 0 | 0 | 0 | 2 |
| 0 | | 1 | 2 | 0 | 0 | 1 | 2 | 0 | 0 | 0 | 0 | 0 | 2 |
| 0 | | 1 | 2 | 0 | 0 | 1 | 2 | 0 | 0 | 0 | 0 | 0 | 2 |
| 0 | | 1 | 2 | 0 | 0 | 1 | 2 | 0 | 0 | 0 | 0 | 0 | 2 |
| 1 |
| --- |
| i1 |
| --- |
| 2 |
| --- |
| 3 |
| --- |
| 4 |
| --- |
| 5 |
| --- |
| 6 |
| --- |
| 7 |
| --- |
| 8 |
| --- |
| 9 |
| --- |
| 10 |
| --- |
| 11 |
| --- |
| 12 |
| --- |
| 13 |
| --- |
Class I
Class IA
Class IB
Class IIA
Class II
Class IIB
Figure S2. Evolution of intron gain and loss events of the plant PHO1 gene family.
