# Supplementary figures and images for: Phylogeny, structural evolution and functional diversification of the plant PHOSPHATE1 gene family: a focus on Glycine max
Source: BMC Evol Biol. 2013 May 24;13:103. doi: 10.1186/1471-2148-13-103 (PMC3680083; doi:10.1186/1471-2148-13-103)

## Slide 1
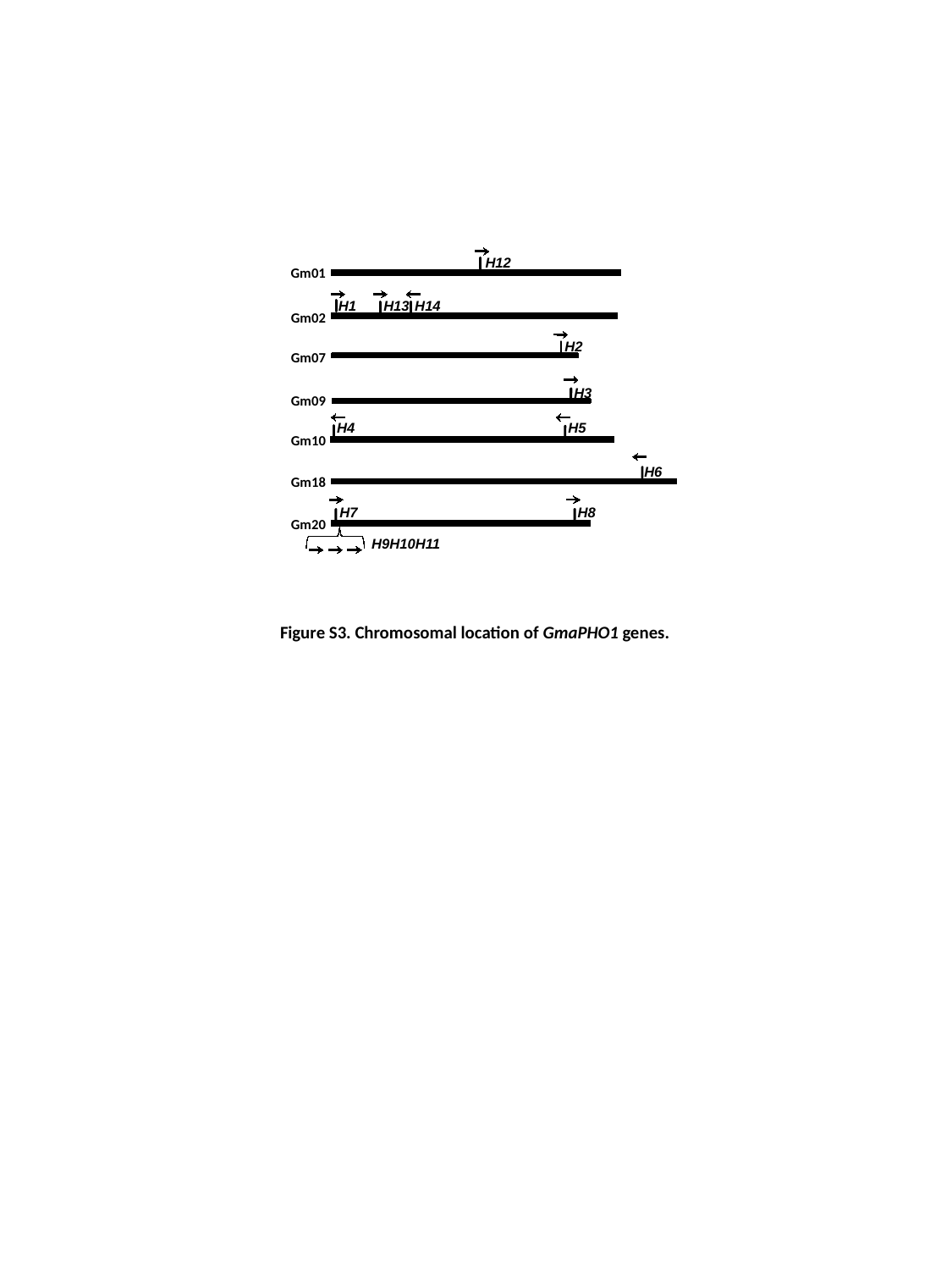

H12
Gm01
H14
H13
H1
Gm02
H2
Gm07
H3
Gm09
H4
H5
Gm10
H6
Gm18
H7
H8
Gm20
H9H10H11
Figure S3. Chromosomal location of GmaPHO1 genes.

Supplement: Additional file 7: Figure S3 — Chromosomal location of GmaPHO1 genes. The horizontal line represents the chromosome and the vertical lines represent the GmaPHO1 genes. Arrows indicate the transcriptional directions of the corresponding genes. H1-H14 are abbreviated for GmaPHO1; H1-H14 genes. [file 1471-2148-13-103-S7.pptx]

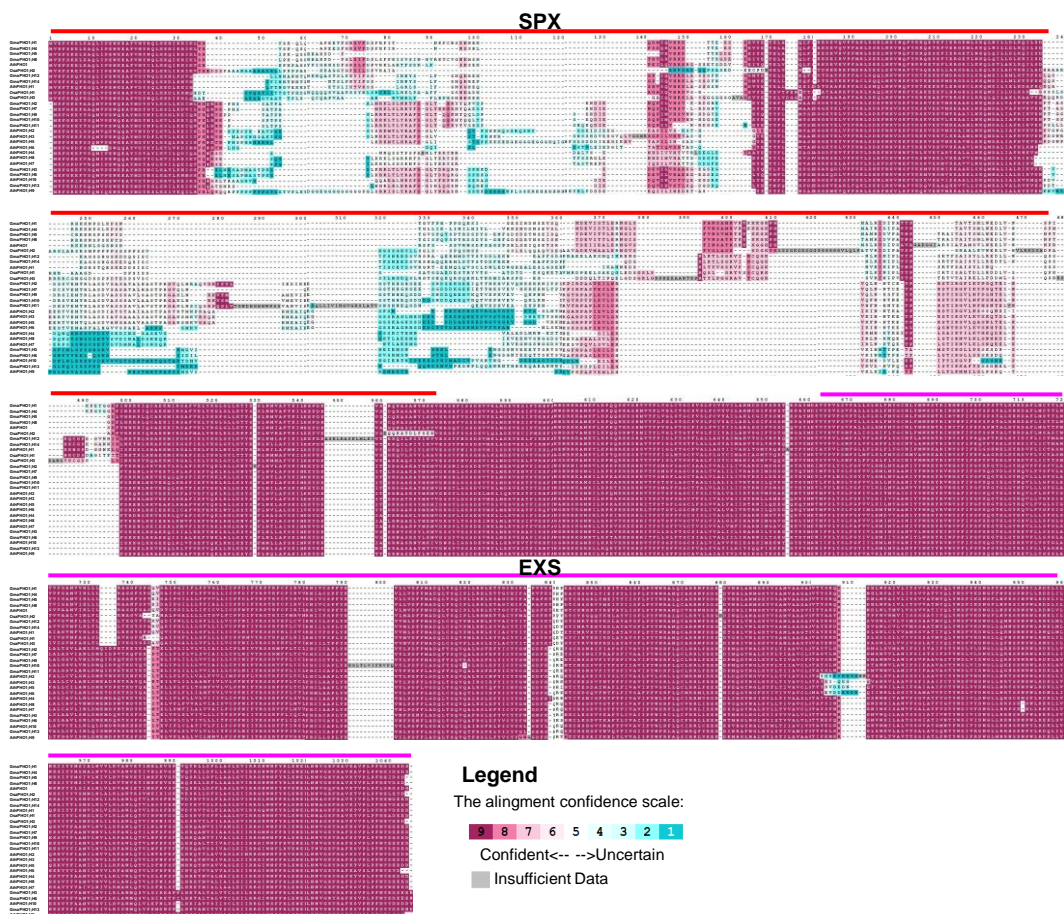

**Figure S4. Multiple sequence alignment of PHO1 proteins in soybean, *Arabidopsis* and rice.**

Supplement: Additional file 9: Figure S4 — Multiple sequence alignment of PHO1 proteins in soybean, Arabidopsis and rice. The characteristic domains of the PHO1 proteins are highlighted in red (SPX, Pfam PF03105) and pink (EXS, Pfam PF03124) lines, respectively. [file 1471-2148-13-103-S9.pdf]
