# Supplementary material for: Phylogeny, structural evolution and functional diversification of the plant PHOSPHATE1 gene family: a focus on Glycine max
Source: BMC Evol Biol. 2013 May 24;13:103. doi: 10.1186/1471-2148-13-103 (PMC3680083; doi:10.1186/1471-2148-13-103)
Supplement: Additional file 8: Table S5 — Variation in exon number and length of GmaPHO1 genes. [file 1471-2148-13-103-S8.pptx]

## Slide 1
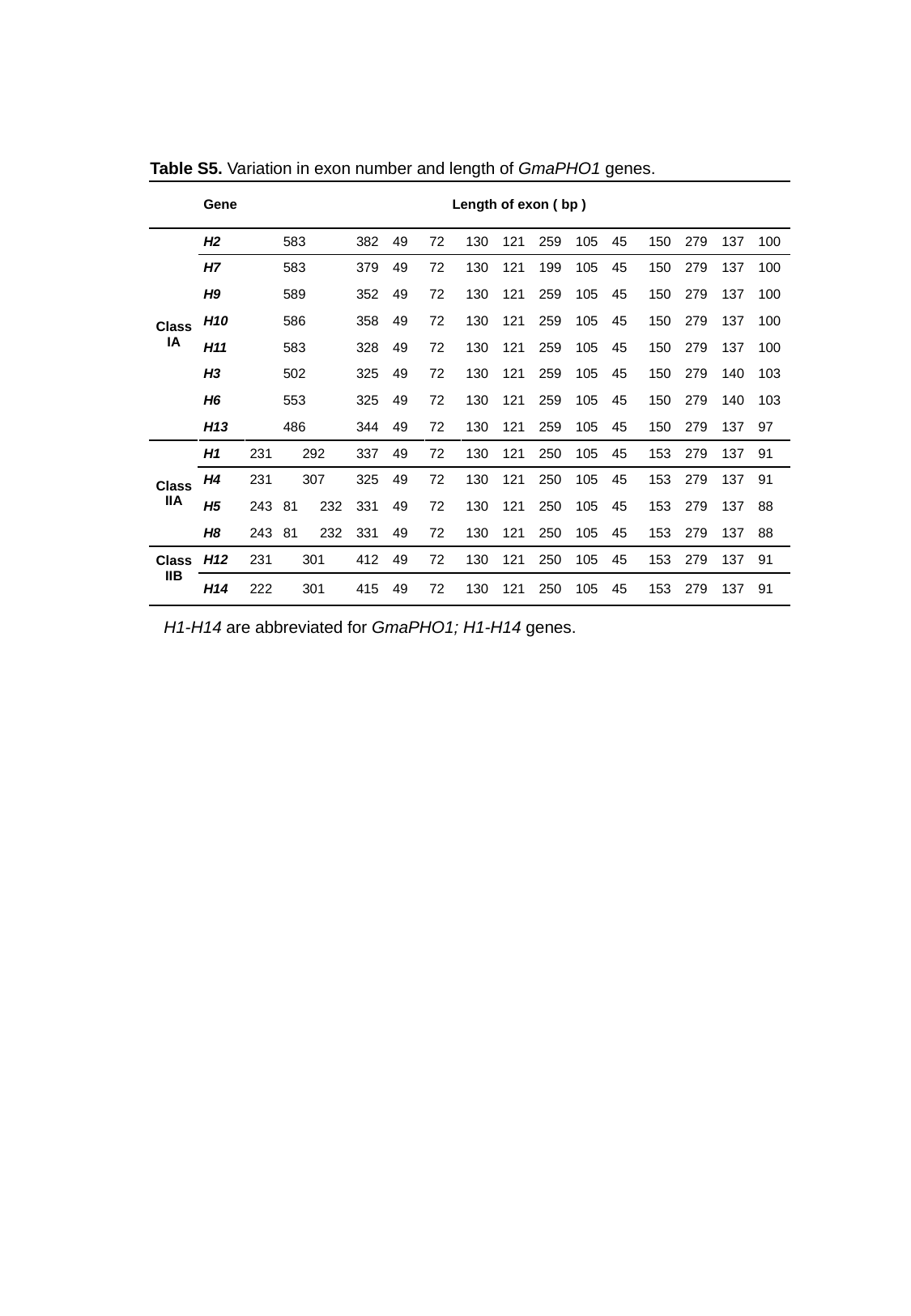

Table S5. Variation in exon number and length of GmaPHO1 genes.
| | Gene | Length of exon ( bp ) | | | | | | | | | | | | | | |
| --- | --- | --- | --- | --- | --- | --- | --- | --- | --- | --- | --- | --- | --- | --- | --- | --- |
| Class IA | H2 | | 583 | | 382 | 49 | 72 | 130 | 121 | 259 | 105 | 45 | 150 | 279 | 137 | 100 |
| | H7 | | 583 | | 379 | 49 | 72 | 130 | 121 | 199 | 105 | 45 | 150 | 279 | 137 | 100 |
| | H9 | | 589 | | 352 | 49 | 72 | 130 | 121 | 259 | 105 | 45 | 150 | 279 | 137 | 100 |
| | H10 | | 586 | | 358 | 49 | 72 | 130 | 121 | 259 | 105 | 45 | 150 | 279 | 137 | 100 |
| | H11 | | 583 | | 328 | 49 | 72 | 130 | 121 | 259 | 105 | 45 | 150 | 279 | 137 | 100 |
| | H3 | | 502 | | 325 | 49 | 72 | 130 | 121 | 259 | 105 | 45 | 150 | 279 | 140 | 103 |
| | H6 | | 553 | | 325 | 49 | 72 | 130 | 121 | 259 | 105 | 45 | 150 | 279 | 140 | 103 |
| | H13 | | 486 | | 344 | 49 | 72 | 130 | 121 | 259 | 105 | 45 | 150 | 279 | 137 | 97 |
| ClassIIA | H1 | 231 | 292 | | 337 | 49 | 72 | 130 | 121 | 250 | 105 | 45 | 153 | 279 | 137 | 91 |
| | H4 | 231 | 307 | | 325 | 49 | 72 | 130 | 121 | 250 | 105 | 45 | 153 | 279 | 137 | 91 |
| | H5 | 243 | 81 | 232 | 331 | 49 | 72 | 130 | 121 | 250 | 105 | 45 | 153 | 279 | 137 | 88 |
| | H8 | 243 | 81 | 232 | 331 | 49 | 72 | 130 | 121 | 250 | 105 | 45 | 153 | 279 | 137 | 88 |
| Class IIB | H12 | 231 | 301 | | 412 | 49 | 72 | 130 | 121 | 250 | 105 | 45 | 153 | 279 | 137 | 91 |
| | H14 | 222 | 301 | | 415 | 49 | 72 | 130 | 121 | 250 | 105 | 45 | 153 | 279 | 137 | 91 |
H1-H14 are abbreviated for GmaPHO1; H1-H14 genes.
