# Supplementary material for: Phylogeny, structural evolution and functional diversification of the plant PHOSPHATE1 gene family: a focus on Glycine max
Source: BMC Evol Biol. 2013 May 24;13:103. doi: 10.1186/1471-2148-13-103 (PMC3680083; doi:10.1186/1471-2148-13-103)
Supplement: Additional file 10: Table S6 — Pairwise comparison of the GmaPHO1 protein family. [file 1471-2148-13-103-S10.pptx]

## Slide 1
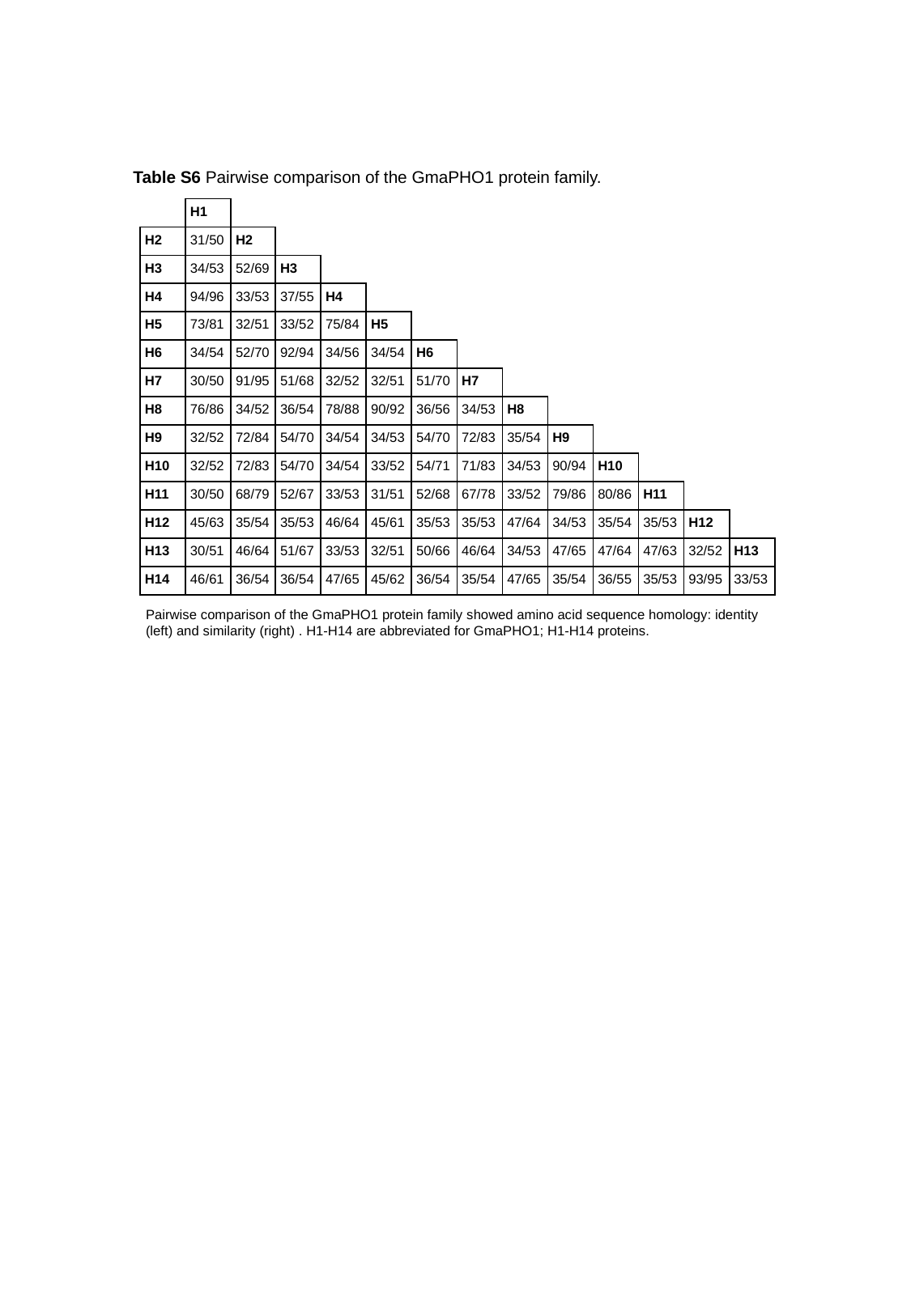

Table S6 Pairwise comparison of the GmaPHO1 protein family.
| | H1 | | | | | | | | | | | | |
| --- | --- | --- | --- | --- | --- | --- | --- | --- | --- | --- | --- | --- | --- |
| H2 | 31/50 | H2 | | | | | | | | | | | |
| H3 | 34/53 | 52/69 | H3 | | | | | | | | | | |
| H4 | 94/96 | 33/53 | 37/55 | H4 | | | | | | | | | |
| H5 | 73/81 | 32/51 | 33/52 | 75/84 | H5 | | | | | | | | |
| H6 | 34/54 | 52/70 | 92/94 | 34/56 | 34/54 | H6 | | | | | | | |
| H7 | 30/50 | 91/95 | 51/68 | 32/52 | 32/51 | 51/70 | H7 | | | | | | |
| H8 | 76/86 | 34/52 | 36/54 | 78/88 | 90/92 | 36/56 | 34/53 | H8 | | | | | |
| H9 | 32/52 | 72/84 | 54/70 | 34/54 | 34/53 | 54/70 | 72/83 | 35/54 | H9 | | | | |
| H10 | 32/52 | 72/83 | 54/70 | 34/54 | 33/52 | 54/71 | 71/83 | 34/53 | 90/94 | H10 | | | |
| H11 | 30/50 | 68/79 | 52/67 | 33/53 | 31/51 | 52/68 | 67/78 | 33/52 | 79/86 | 80/86 | H11 | | |
| H12 | 45/63 | 35/54 | 35/53 | 46/64 | 45/61 | 35/53 | 35/53 | 47/64 | 34/53 | 35/54 | 35/53 | H12 | |
| H13 | 30/51 | 46/64 | 51/67 | 33/53 | 32/51 | 50/66 | 46/64 | 34/53 | 47/65 | 47/64 | 47/63 | 32/52 | H13 |
| H14 | 46/61 | 36/54 | 36/54 | 47/65 | 45/62 | 36/54 | 35/54 | 47/65 | 35/54 | 36/55 | 35/53 | 93/95 | 33/53 |
Pairwise comparison of the GmaPHO1 protein family showed amino acid sequence homology: identity (left) and similarity (right) . H1-H14 are abbreviated for GmaPHO1; H1-H14 proteins.
