# Supplementary material for: Phylogeny, structural evolution and functional diversification of the plant PHOSPHATE1 gene family: a focus on Glycine max
Source: BMC Evol Biol. 2013 May 24;13:103. doi: 10.1186/1471-2148-13-103 (PMC3680083; doi:10.1186/1471-2148-13-103)
Supplement: Additional file 11: Table S7 — Primer sequences of GmaPHO1 genes used in the present work. [file 1471-2148-13-103-S11.pptx]

## Slide 1
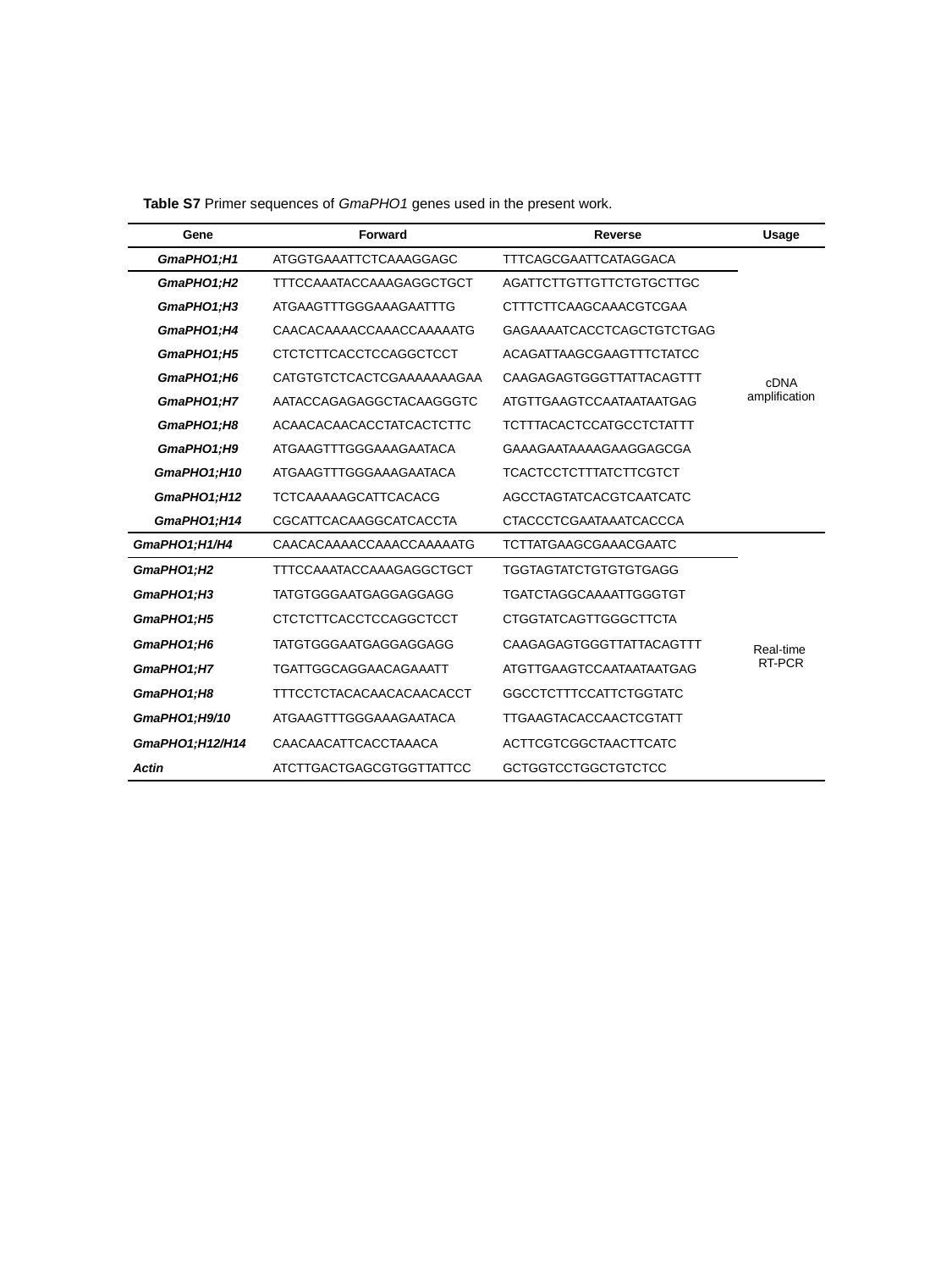

Table S7 Primer sequences of GmaPHO1 genes used in the present work.
| Gene | Forward | Reverse | Usage |
| --- | --- | --- | --- |
| GmaPHO1;H1 | ATGGTGAAATTCTCAAAGGAGC | TTTCAGCGAATTCATAGGACA | cDNA amplification |
| GmaPHO1;H2 | TTTCCAAATACCAAAGAGGCTGCT | AGATTCTTGTTGTTCTGTGCTTGC | |
| GmaPHO1;H3 | ATGAAGTTTGGGAAAGAATTTG | CTTTCTTCAAGCAAACGTCGAA | |
| GmaPHO1;H4 | CAACACAAAACCAAACCAAAAATG | GAGAAAATCACCTCAGCTGTCTGAG | |
| GmaPHO1;H5 | CTCTCTTCACCTCCAGGCTCCT | ACAGATTAAGCGAAGTTTCTATCC | |
| GmaPHO1;H6 | CATGTGTCTCACTCGAAAAAAAGAA | CAAGAGAGTGGGTTATTACAGTTT | |
| GmaPHO1;H7 | AATACCAGAGAGGCTACAAGGGTC | ATGTTGAAGTCCAATAATAATGAG | |
| GmaPHO1;H8 | ACAACACAACACCTATCACTCTTC | TCTTTACACTCCATGCCTCTATTT | |
| GmaPHO1;H9 | ATGAAGTTTGGGAAAGAATACA | GAAAGAATAAAAGAAGGAGCGA | |
| GmaPHO1;H10 | ATGAAGTTTGGGAAAGAATACA | TCACTCCTCTTTATCTTCGTCT | |
| GmaPHO1;H12 | TCTCAAAAAGCATTCACACG | AGCCTAGTATCACGTCAATCATC | |
| GmaPHO1;H14 | CGCATTCACAAGGCATCACCTA | CTACCCTCGAATAAATCACCCA | |
| GmaPHO1;H1/H4 | CAACACAAAACCAAACCAAAAATG | TCTTATGAAGCGAAACGAATC | Real-time RT-PCR |
| GmaPHO1;H2 | TTTCCAAATACCAAAGAGGCTGCT | TGGTAGTATCTGTGTGTGAGG | |
| GmaPHO1;H3 | TATGTGGGAATGAGGAGGAGG | TGATCTAGGCAAAATTGGGTGT | |
| GmaPHO1;H5 | CTCTCTTCACCTCCAGGCTCCT | CTGGTATCAGTTGGGCTTCTA | |
| GmaPHO1;H6 | TATGTGGGAATGAGGAGGAGG | CAAGAGAGTGGGTTATTACAGTTT | |
| GmaPHO1;H7 | TGATTGGCAGGAACAGAAATT | ATGTTGAAGTCCAATAATAATGAG | |
| GmaPHO1;H8 | TTTCCTCTACACAACACAACACCT | GGCCTCTTTCCATTCTGGTATC | |
| GmaPHO1;H9/10 | ATGAAGTTTGGGAAAGAATACA | TTGAAGTACACCAACTCGTATT | |
| GmaPHO1;H12/H14 | CAACAACATTCACCTAAACA | ACTTCGTCGGCTAACTTCATC | |
| Actin | ATCTTGACTGAGCGTGGTTATTCC | GCTGGTCCTGGCTGTCTCC | |
