# Supplementary material for: Phylogeny, structural evolution and functional diversification of the plant PHOSPHATE1 gene family: a focus on Glycine max
Source: BMC Evol Biol. 2013 May 24;13:103. doi: 10.1186/1471-2148-13-103 (PMC3680083; doi:10.1186/1471-2148-13-103)
Supplement: Additional files 12: Figure S5 — The reciprocal significance of gene expression variation among tissues. (a-e) Significance of organ-specific expressions the Class I genes. (f-i) Significance of organ-specific expressions of the Class II genes. [file 1471-2148-13-103-S12.pptx]

## Slide 1
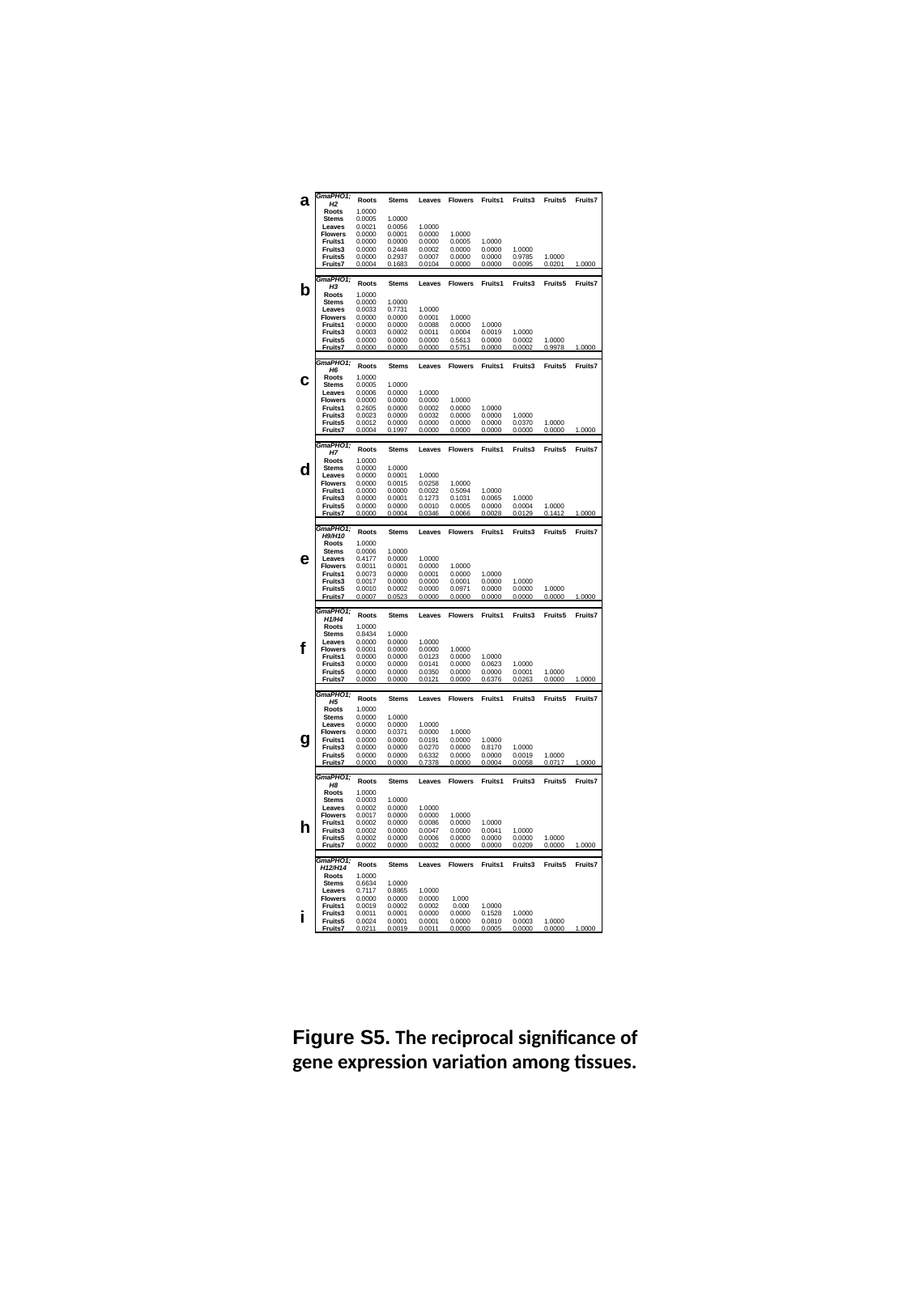

a
| GmaPHO1; H2 | Roots | Stems | Leaves | Flowers | Fruits1 | Fruits3 | Fruits5 | Fruits7 |
| --- | --- | --- | --- | --- | --- | --- | --- | --- |
| Roots | 1.0000 | | | | | | | |
| Stems | 0.0005 | 1.0000 | | | | | | |
| Leaves | 0.0021 | 0.0056 | 1.0000 | | | | | |
| Flowers | 0.0000 | 0.0001 | 0.0000 | 1.0000 | | | | |
| Fruits1 | 0.0000 | 0.0000 | 0.0000 | 0.0005 | 1.0000 | | | |
| Fruits3 | 0.0000 | 0.2448 | 0.0002 | 0.0000 | 0.0000 | 1.0000 | | |
| Fruits5 | 0.0000 | 0.2937 | 0.0007 | 0.0000 | 0.0000 | 0.9785 | 1.0000 | |
| Fruits7 | 0.0004 | 0.1683 | 0.0104 | 0.0000 | 0.0000 | 0.0095 | 0.0201 | 1.0000 |
| | | | | | | | | |
| GmaPHO1; H3 | Roots | Stems | Leaves | Flowers | Fruits1 | Fruits3 | Fruits5 | Fruits7 |
| Roots | 1.0000 | | | | | | | |
| Stems | 0.0000 | 1.0000 | | | | | | |
| Leaves | 0.0033 | 0.7731 | 1.0000 | | | | | |
| Flowers | 0.0000 | 0.0000 | 0.0001 | 1.0000 | | | | |
| Fruits1 | 0.0000 | 0.0000 | 0.0088 | 0.0000 | 1.0000 | | | |
| Fruits3 | 0.0003 | 0.0002 | 0.0011 | 0.0004 | 0.0019 | 1.0000 | | |
| Fruits5 | 0.0000 | 0.0000 | 0.0000 | 0.5613 | 0.0000 | 0.0002 | 1.0000 | |
| Fruits7 | 0.0000 | 0.0000 | 0.0000 | 0.5751 | 0.0000 | 0.0002 | 0.9978 | 1.0000 |
| | | | | | | | | |
| GmaPHO1; H6 | Roots | Stems | Leaves | Flowers | Fruits1 | Fruits3 | Fruits5 | Fruits7 |
| Roots | 1.0000 | | | | | | | |
| Stems | 0.0005 | 1.0000 | | | | | | |
| Leaves | 0.0006 | 0.0000 | 1.0000 | | | | | |
| Flowers | 0.0000 | 0.0000 | 0.0000 | 1.0000 | | | | |
| Fruits1 | 0.2605 | 0.0000 | 0.0002 | 0.0000 | 1.0000 | | | |
| Fruits3 | 0.0023 | 0.0000 | 0.0032 | 0.0000 | 0.0000 | 1.0000 | | |
| Fruits5 | 0.0012 | 0.0000 | 0.0000 | 0.0000 | 0.0000 | 0.0370 | 1.0000 | |
| Fruits7 | 0.0004 | 0.1997 | 0.0000 | 0.0000 | 0.0000 | 0.0000 | 0.0000 | 1.0000 |
| | | | | | | | | |
| GmaPHO1; H7 | Roots | Stems | Leaves | Flowers | Fruits1 | Fruits3 | Fruits5 | Fruits7 |
| Roots | 1.0000 | | | | | | | |
| Stems | 0.0000 | 1.0000 | | | | | | |
| Leaves | 0.0000 | 0.0001 | 1.0000 | | | | | |
| Flowers | 0.0000 | 0.0015 | 0.0258 | 1.0000 | | | | |
| Fruits1 | 0.0000 | 0.0000 | 0.0022 | 0.5094 | 1.0000 | | | |
| Fruits3 | 0.0000 | 0.0001 | 0.1273 | 0.1031 | 0.0065 | 1.0000 | | |
| Fruits5 | 0.0000 | 0.0000 | 0.0010 | 0.0005 | 0.0000 | 0.0004 | 1.0000 | |
| Fruits7 | 0.0000 | 0.0004 | 0.0346 | 0.0066 | 0.0028 | 0.0129 | 0.1412 | 1.0000 |
| | | | | | | | | |
| GmaPHO1; H9/H10 | Roots | Stems | Leaves | Flowers | Fruits1 | Fruits3 | Fruits5 | Fruits7 |
| Roots | 1.0000 | | | | | | | |
| Stems | 0.0006 | 1.0000 | | | | | | |
| Leaves | 0.4177 | 0.0000 | 1.0000 | | | | | |
| Flowers | 0.0011 | 0.0001 | 0.0000 | 1.0000 | | | | |
| Fruits1 | 0.0073 | 0.0000 | 0.0001 | 0.0000 | 1.0000 | | | |
| Fruits3 | 0.0017 | 0.0000 | 0.0000 | 0.0001 | 0.0000 | 1.0000 | | |
| Fruits5 | 0.0010 | 0.0002 | 0.0000 | 0.0971 | 0.0000 | 0.0000 | 1.0000 | |
| Fruits7 | 0.0007 | 0.0523 | 0.0000 | 0.0000 | 0.0000 | 0.0000 | 0.0000 | 1.0000 |
| | | | | | | | | |
| GmaPHO1; H1/H4 | Roots | Stems | Leaves | Flowers | Fruits1 | Fruits3 | Fruits5 | Fruits7 |
| Roots | 1.0000 | | | | | | | |
| Stems | 0.8434 | 1.0000 | | | | | | |
| Leaves | 0.0000 | 0.0000 | 1.0000 | | | | | |
| Flowers | 0.0001 | 0.0000 | 0.0000 | 1.0000 | | | | |
| Fruits1 | 0.0000 | 0.0000 | 0.0123 | 0.0000 | 1.0000 | | | |
| Fruits3 | 0.0000 | 0.0000 | 0.0141 | 0.0000 | 0.0623 | 1.0000 | | |
| Fruits5 | 0.0000 | 0.0000 | 0.0350 | 0.0000 | 0.0000 | 0.0001 | 1.0000 | |
| Fruits7 | 0.0000 | 0.0000 | 0.0121 | 0.0000 | 0.6376 | 0.0263 | 0.0000 | 1.0000 |
| | | | | | | | | |
| GmaPHO1; H5 | Roots | Stems | Leaves | Flowers | Fruits1 | Fruits3 | Fruits5 | Fruits7 |
| Roots | 1.0000 | | | | | | | |
| Stems | 0.0000 | 1.0000 | | | | | | |
| Leaves | 0.0000 | 0.0000 | 1.0000 | | | | | |
| Flowers | 0.0000 | 0.0371 | 0.0000 | 1.0000 | | | | |
| Fruits1 | 0.0000 | 0.0000 | 0.0191 | 0.0000 | 1.0000 | | | |
| Fruits3 | 0.0000 | 0.0000 | 0.0270 | 0.0000 | 0.8170 | 1.0000 | | |
| Fruits5 | 0.0000 | 0.0000 | 0.6332 | 0.0000 | 0.0000 | 0.0019 | 1.0000 | |
| Fruits7 | 0.0000 | 0.0000 | 0.7378 | 0.0000 | 0.0004 | 0.0058 | 0.0717 | 1.0000 |
| | | | | | | | | |
| GmaPHO1; H8 | Roots | Stems | Leaves | Flowers | Fruits1 | Fruits3 | Fruits5 | Fruits7 |
| Roots | 1.0000 | | | | | | | |
| Stems | 0.0003 | 1.0000 | | | | | | |
| Leaves | 0.0002 | 0.0000 | 1.0000 | | | | | |
| Flowers | 0.0017 | 0.0000 | 0.0000 | 1.0000 | | | | |
| Fruits1 | 0.0002 | 0.0000 | 0.0086 | 0.0000 | 1.0000 | | | |
| Fruits3 | 0.0002 | 0.0000 | 0.0047 | 0.0000 | 0.0041 | 1.0000 | | |
| Fruits5 | 0.0002 | 0.0000 | 0.0006 | 0.0000 | 0.0000 | 0.0000 | 1.0000 | |
| Fruits7 | 0.0002 | 0.0000 | 0.0032 | 0.0000 | 0.0000 | 0.0209 | 0.0000 | 1.0000 |
| | | | | | | | | |
| GmaPHO1; H12/H14 | Roots | Stems | Leaves | Flowers | Fruits1 | Fruits3 | Fruits5 | Fruits7 |
| Roots | 1.0000 | | | | | | | |
| Stems | 0.6634 | 1.0000 | | | | | | |
| Leaves | 0.7117 | 0.8865 | 1.0000 | | | | | |
| Flowers | 0.0000 | 0.0000 | 0.0000 | 1.000 | | | | |
| Fruits1 | 0.0019 | 0.0002 | 0.0002 | 0.000 | 1.0000 | | | |
| Fruits3 | 0.0011 | 0.0001 | 0.0000 | 0.0000 | 0.1528 | 1.0000 | | |
| Fruits5 | 0.0024 | 0.0001 | 0.0001 | 0.0000 | 0.0810 | 0.0003 | 1.0000 | |
| Fruits7 | 0.0211 | 0.0019 | 0.0011 | 0.0000 | 0.0005 | 0.0000 | 0.0000 | 1.0000 |
b
c
d
e
f
g
h
i
Figure S5. The reciprocal significance of gene expression variation among tissues.
