# Supplementary material for: Phylogeny, structural evolution and functional diversification of the plant PHOSPHATE1 gene family: a focus on Glycine max
Source: BMC Evol Biol. 2013 May 24;13:103. doi: 10.1186/1471-2148-13-103 (PMC3680083; doi:10.1186/1471-2148-13-103)
Supplement: Additional files 13: Figure S6 — Expression of GmaPHO1 genes under Pi deficient or sufficient treatments. (a-d) Root expression of the Class I genes in response to Pi stresses. (e-h) Root expression of the Class II genes in response to Pi stresses. The gene is indicated in each graph. Light grey lines represent the untreated controls; dark gray lines represent the treatment of LP-HP; black lines represent the treatment of HP-LP (see Methods). Actin was used as an internal control. The experiments were performed based on three independent biological samples. Error bars=standard deviations. Asterisks indicate significance of gene expression variation under each treatment in comparison to the untreated control (*P < 0.05, **P < 0.01 and ***P < 0.001). [file 1471-2148-13-103-S13.pptx]

## Slide 1
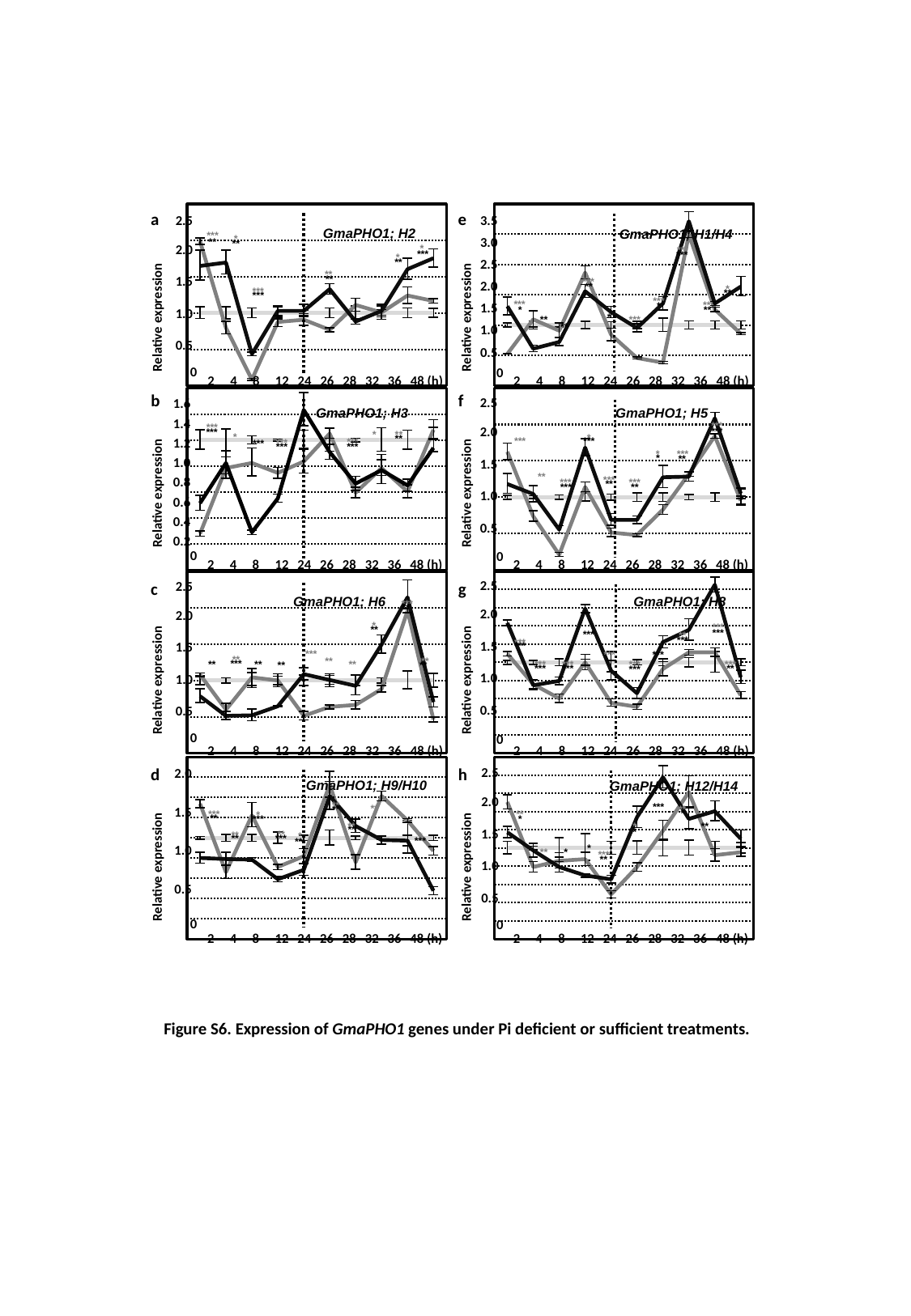

### Chart
| Category | | | |
|---|---|---|---|
| 2 | 1.0 | 1.9861849908740707 | 1.6471820345351549 |
| 4 | 1.0 | 0.7955364837549121 | 1.693490624725054 |
| 8 | 1.0 | 0.08420209855410556 | 0.43527528164806434 |
| 12 | 1.0 | 0.8766057213160356 | 1.0281138266560792 |
| 24 | 1.0 | 0.9075191553171563 | 1.0281138266560805 |
| 26 | 1.0 | 0.7684375906440056 | 1.3286858140965245 |
| 28 | 1.0 | 1.1095694720678366 | 0.8827029962906555 |
| 32 | 1.0 | 1.0069555500567264 | 1.028113826656078 |
| 36 | 1.0 | 1.2397076999389858 | 1.6021397551792438 |
| 48 | 1.0 | 1.164733586468458 | 1.7532114426320606 |
### Chart
| Category | | | |
|---|---|---|---|
| 2 | 1.0 | 0.5248583418115326 | 1.3103934038583647 |
| 4 | 1.0 | 1.09429370126074 | 0.6113201388460348 |
| 8 | 1.0 | 0.9075191553171554 | 0.721964597761244 |
| 12 | 1.0 | 1.8660659830736201 | 1.5583291593209978 |
| 24 | 1.0 | 0.8408964152537209 | 1.2141948843950494 |
| 26 | 1.0 | 0.45691572511470213 | 0.9526379980439377 |
| 28 | 1.0 | 0.38156480224014344 | 1.3566043274476698 |
| 32 | 1.0 | 2.4966610978032167 | 2.7132086548953436 |
| 36 | 1.0 | 1.2570133745218321 | 1.3566043274476698 |
| 48 | 1.0 | 0.8585654364377547 | 1.635804117115556 |a
e
3.5
2.5
GmaPHO1; H2
GmaPHO1; H1/H4
3.0
2.0
2.5
1.5
2.0
Relative expression
Relative expression
1.5
1.0
1.0
0.5
0.5
0
0
2
4
8
12
24
26
28
32
36
48
(h)
2
4
8
12
24
26
28
32
36
48
(h)
b
f
### Chart
| Category | | | |
|---|---|---|---|
| 2 | 1.0 | 0.279321784518057 | 0.5140569133280336 |
| 4 | 1.0 | 0.7845840978967459 | 0.8235910172675825 |
| 8 | 1.0 | 0.8235910172675805 | 0.289172045976322 |
| 12 | 1.0 | 0.7474246243174693 | 0.5509525579383049 |
| 24 | 1.0 | 0.835087919428375 | 1.2311444133449068 |
| 26 | 1.0 | 1.0497166836230658 | 0.9075191553171572 |
| 28 | 1.0 | 0.5946035575013613 | 0.6643429070482566 |
| 32 | 1.0 | 0.7845840978967459 | 0.7684375906440049 |
| 36 | 1.0 | 0.6029039138453837 | 0.6506709277209671 |
| 48 | 1.0 | 1.0792282365044206 | 0.9395227492140116 |
### Chart
| Category | | | |
|---|---|---|---|
| 2 | 1.0 | 1.6245047927124583 | 1.180992661429532 |
| 4 | 1.0 | 0.7371346086455558 | 1.042465760841122 |
| 8 | 0.9999999999999984 | 0.21022410381342974 | 0.5547847360339226 |
| 12 | 1.0 | 1.1407637158684238 | 1.6817928305074286 |
| 24 | 1.0 | 0.5140569133280336 | 0.6877709090698781 |
| 26 | 1.0 | 0.47963205966263217 | 0.6877709090698768 |
| 28 | 1.0 | 0.8293195458144416 | 1.2745606273192618 |
| 32 | 1.0 | 1.3195079107728942 | 1.283425897562893 |
| 36 | 1.0 | 1.8403753012497501 | 2.0849315216822633 |
| 48 | 1.0 | 0.9526379980439393 | 1.0497166836230658 |2.5
1.6
GmaPHO1; H5
GmaPHO1; H3
1.4
2.0
1.2
1.0
1.5
0.8
Relative expression
Relative expression
1.0
0.6
0.4
0.5
0.2
0
0
2
4
8
12
24
26
28
32
36
48
(h)
2
4
8
12
24
26
28
32
36
48
(h)
### Chart
| Category | | | |
|---|---|---|---|
| 2 | 1.0 | 1.0792282365044177 | 0.7845840978967459 |
| 4 | 1.0 | 0.5987393523094646 | 0.5140569133280339 |
| 8 | 1.0 | 1.042465760841122 | 0.5212328804205605 |
| 12 | 1.0 | 1.0 | 0.6461764153187513 |
| 24 | 1.0 | 0.5140569133280339 | 1.0867348625260609 |
| 26 | 1.0 | 0.6372803136596342 | 1.0069555500567284 |
| 28 | 1.0 | 0.6643429070482558 | 0.9265880618903706 |
| 32 | 1.0 | 0.8827029962906535 | 1.4948492486349279 |
| 36 | 1.0 | 1.9588405951738541 | 2.1435469250725845 |
| 48 | 1.0 | 0.4632940309451853 | 0.7022224378689979 |
### Chart
| Category | | | |
|---|---|---|---|
| 2 | 1.0 | 1.09429370126074 | 1.4339552480158198 |
| 4 | 1.0 | 0.7578582832552034 | 0.7474246243174689 |
| 8 | 1.0 | 0.6029039138453854 | 0.7955364837549131 |
| 12 | 1.0 | 1.0 | 1.5910729675098363 |
| 24 | 1.0 | 0.5547847360339228 | 0.9075191553171573 |
| 26 | 1.0 | 0.5105060628535967 | 0.6597539553864477 |
| 28 | 1.0 | 0.9265880618903716 | 1.2226402776920613 |
| 32 | 1.0 | 1.1095694720678366 | 1.3566043274476698 |
| 36 | 1.0 | 1.109569472067836 | 1.8531761237807527 |
| 48 | 1.0 | 0.6372803136596354 | 0.8350879194283697 |2.5
2.5
c
g
GmaPHO1; H6
GmaPHO1; H8
2.0
2.0
1.5
1.5
Relative expression
Relative expression
1.0
1.0
0.5
0.5
0
0
2
4
8
12
24
26
28
32
36
48
(h)
2
4
8
12
24
26
28
32
36
48
(h)
### Chart
| Category | | | |
|---|---|---|---|
| 2 | 1.0 | 1.33792755478611 | 0.8010698775896261 |
| 4 | 1.0 | 0.6597539553864477 | 0.7900413118633831 |
| 8 | 1.0 | 1.2226402776920606 | 0.7845840978967463 |
| 12 | 1.0 | 0.7120250977985415 | 0.5904963307147594 |
| 24 | 1.0 | 0.8179020585577818 | 0.6830201283771998 |
| 26 | 1.0 | 1.5262592089605584 | 1.4142135623730951 |
| 28 | 1.0 | 0.7578582832552042 | 1.1172871380722302 |
| 32 | 1.0 | 1.414213562373096 | 0.9794202975869266 |
| 36 | 1.0 | 1.1728349492318781 | 0.9726549474122866 |
| 48 | 1.0 | 0.8705505632961223 | 0.47631899902197267 |
### Chart
| Category | | | |
|---|---|---|---|
| 2 | 1.0 | 1.5052467474110638 | 1.1728349492318801 |
| 4 | 1.0 | 0.7900413118633812 | 0.9726549474122856 |
| 8 | 1.0 | 0.8585654364377556 | 0.7900413118633804 |
| 12 | 1.0 | 0.8766057213160356 | 0.6973718331752082 |
| 24 | 1.0 | 0.4897101487934641 | 0.655196701929186 |
| 26 | 1.0 | 0.7900413118633819 | 1.337927554786114 |
| 28 | 1.0 | 1.1892071150027221 | 1.7776853623331437 |
| 32 | 1.0 | 1.6132835184442529 | 1.3195079107728953 |
| 36 | 1.0 | 0.9201876506248803 | 1.4044448757379902 |
| 48 | 1.0 | 0.9526379980439371 | 1.101905115876611 |d
h
2.5
2.0
GmaPHO1; H9/H10
GmaPHO1; H12/H14
2.0
1.5
1.5
1.0
Relative expression
Relative expression
1.0
0.5
0.5
0
0
2
4
12
24
26
28
32
36
48
(h)
2
4
8
12
24
26
28
32
36
48
(h)
8
***
*
**
**
*
***
***
***
*
**
**
**
***
**
*
***
**
***
***
***
**
*
*
**
*
**
***
**
***
***
***
***
*
**
*
*
**
***
***
***
***
***
***
***
*
***
*
**
**
***
***
***
**
**
***
***
**
*
***
**
***
***
**
***
***
***
***
***
***
**
***
**
***
**
***
***
***
***
**
**
**
**
***
**
***
***
**
***
**
**
**
*
***
**
*
**
***
*
**
*
**
**
*
**
**
*
*
***
**
***
**
*
**
*
***
**
Figure S6. Expression of GmaPHO1 genes under Pi deficient or sufficient treatments.
